# Combined Strategies for Nanodrugs Noninvasively Overcoming the Blood–Brain Barrier and Actively Targeting Glioma Lesions

**DOI:** 10.34133/bmr.0133

**Published:** 2025-02-05

**Authors:** Yuanyuan Liu, Haigang Wu, Gaofeng Liang

**Affiliations:** ^1^ College of Basic Medicine and Forensic Medicine, Henan University of Science and Technology, Luoyang, Henan Province 471000, China.; ^2^ Henan Key Laboratory of Brain Targeted Bio-nanomedicine, School of Life Sciences, Henan University, Kaifeng, Henan Province 475004, China.

## Abstract

Drugs for tumor treatment face various challenges, including poor solubility, poor stability, short blood half-life, nontargeting ability, and strong toxic side effects. Fortunately, nanodrug delivery systems provide excellent solution to these problems. However, nanodrugs for glioma treatment also face some key challenges including overcoming the blood–brain barrier (BBB) and, specifically, accumulation in glioma lesions. In this review, we systematically summarize the advantages and disadvantages of combined strategies for nanodrugs noninvasively overcoming BBB and actively targeting glioma lesions to achieve effective glioma therapy. Common noninvasive strategies for nanodrugs overcoming the BBB include bypassing the BBB via the nose-to-brain route, opening the tight junction of the BBB by focused ultrasound with microbubbles, and transendothelial cell transport by intact cell loading, ligand decoration, or cell membrane camouflage of nanodrugs. Actively targeting glioma lesions after overcoming the BBB is another key factor helping nanodrugs accurately treat in situ gliomas. This aim can also be achieved by loading nanodrugs into intact cells and modifying ligand or cell membrane fragments on the surface of nanodrugs. Targeting decorated nanodrugs can guarantee precise glioma killing and avoid side effects on normal brain tissues that contribute to the specific recognition of glioma lesions. Furthermore, the challenges and prospects of nanodrugs in clinical glioma treatment are discussed.

## Introduction

Glioma is a kind of prevalent brain tumor that originates from abnormal glial cells in the nervous system, representing about 80% of malignant brain tumors [1]. Gliomas encompass various types, including astrocytoma, ependymomas, and oligodendroglioma, each with distinct cellular origins. According to the World Health Organization (WHO), gliomas are classified into grades I to IV, with grades I and II classified as low grade/malignant, and grades III and IV classified as high grade/malignant [2]. High-grade gliomas are more commanding than low-grade gliomas and have worse prognoses [3,4]. Glioblastoma multiforme (GBM) is the most aggressive form and accounts for 60% to 70% of all gliomas [5]. It can manifest either as primary glioblastoma, originating directly in the brain, or as secondary glioblastoma, evolving from lower-grade astrocytomas and anaplastic astrocytomas [6]. The most conventional clinical treatments for glioma include surgery, radiation, chemotherapy, or a combination of these. Surgery is the primary and preferred treatment option for the vast majority of newly diagnosed gliomas, encompassing all subtypes of glioma. After surgery, the adjuvant treatment regimens are administrated based on the specific subtype or status of glioma (Table S1), ensuring a tailored approach for optimal patient outcomes.

Despite undergoing treatment, glioma patients face a challenging prognosis. Approximately 95% of individuals diagnosed with grade I gliomas can expect to survive beyond 5 years. However, grade II gliomas, while generally having a favorable prognosis, can exhibit unpredictable behavior and possess the potential to progress into more aggressive grade III and IV gliomas [7]. Grade III gliomas are characterized by heightened aggressiveness compared to their low-grade counterparts, with a notable risk of recurrence. Grade IV gliomas, commonly known as GBM, exhibit rapid growth and infiltration into healthy brain tissue. Survival rates for GBM are notably low, with only 7% of patients surviving beyond 5 years post-diagnosis. The prognosis is particularly grim, with an average life expectancy ranging from 14 to 16 months [8].

The brain, a vital organ, requires exceptional safeguarding for its preservation and well-being. To shield against germs, infections, and harmful toxins, it possesses a unique security mechanism: the blood–brain barrier (BBB). The BBB, primarily composed of blood vessel endothelial cells (BVECs) along with surrounding vascular cells like astrocytes and pericytes, presents the most important obstacle in this process [9,10]. The primary role of the BBB is to uphold the composition of brain tissue fluid and ensure the homeostasis of the central nervous system (CNS) by finely controlling the passage of diverse molecules between the systemic circulation and the brain interstitial fluid [11]. This protective shield acts like a moat around a castle, allowing only essential nutrients and substances into the brain’s sanctuary. The BBB operates with remarkable efficiency, albeit sometimes posing a hindrance. A pivotal challenge lies in its resilience against permitting the majority of bloodstream medications, including numerous chemotherapy agents, to access the brain. Therefore, devising innovative strategies that enhance drug delivery across this barrier is necessary to optimize the treatment of brain tumors.

In addition to the challenge of penetrating the BBB, there are other inherent limitations of the drugs themselves in the treatment of tumors. Research has demonstrated the efficacy of various drugs, including small molecular chemical drugs, nucleic acid drugs, polypeptide drugs, and protein drugs, in targeting and destroying glioma cells. However, inherent limitations such as hydrophobicity, low stability, restricted cell membrane permeability, lack of targeting specificity, and strong side effects hinder their effective use in glioma treatment [12]. Fortunately, the advent of nanocarrier-mediated drug delivery systems (nanodrugs) offers a promising solution to these challenges. Nanocarriers, including organic, inorganic, and biologically derived varieties, serve to mitigate the aforementioned drawbacks, thereby enhancing the therapeutic potential of drugs for glioma treatment [13].

In the urgent quest for effective treatments for glioma, the timely delivery of nanodrugs is crucial. First, these advanced therapeutic agents must successfully cross the formidable BBB to reach their intended targets. The presence of the BBB is not only a vital protective shield for the brain but also a primary challenge for drug therapy. Whether a drug can successfully navigate this barrier directly determines its ability to reach glioma lesions. Once nanomedicines successfully cross the BBB, they must also specifically target glioma cells to ensure maximum therapeutic efficacy while minimizing damage and side effects to surrounding healthy tissues. This targeting not only enhances treatment efficiency but also reduces the discomfort and complications associated with traditional chemotherapy. Therefore, addressing these 2 critical conditions crossing the BBB and precisely targeting glioma cells are central to achieving optimal therapeutic outcomes in glioma treatment. This information is vital for a broad audience, including medical researchers, oncologists, nanotechnology experts, and patients seeking advanced treatment options. By delving into these requirements, stakeholders can collaborate more effectively to drive the development of innovative nanomedicine therapies for glioma, promoting the research and clinical application of new treatments to improve patient outcomes and quality of life. Enhancing understanding of this complex field can help stimulate more research investment and interdisciplinary collaboration to tackle this marked medical challenge.

**Fig. 1.**
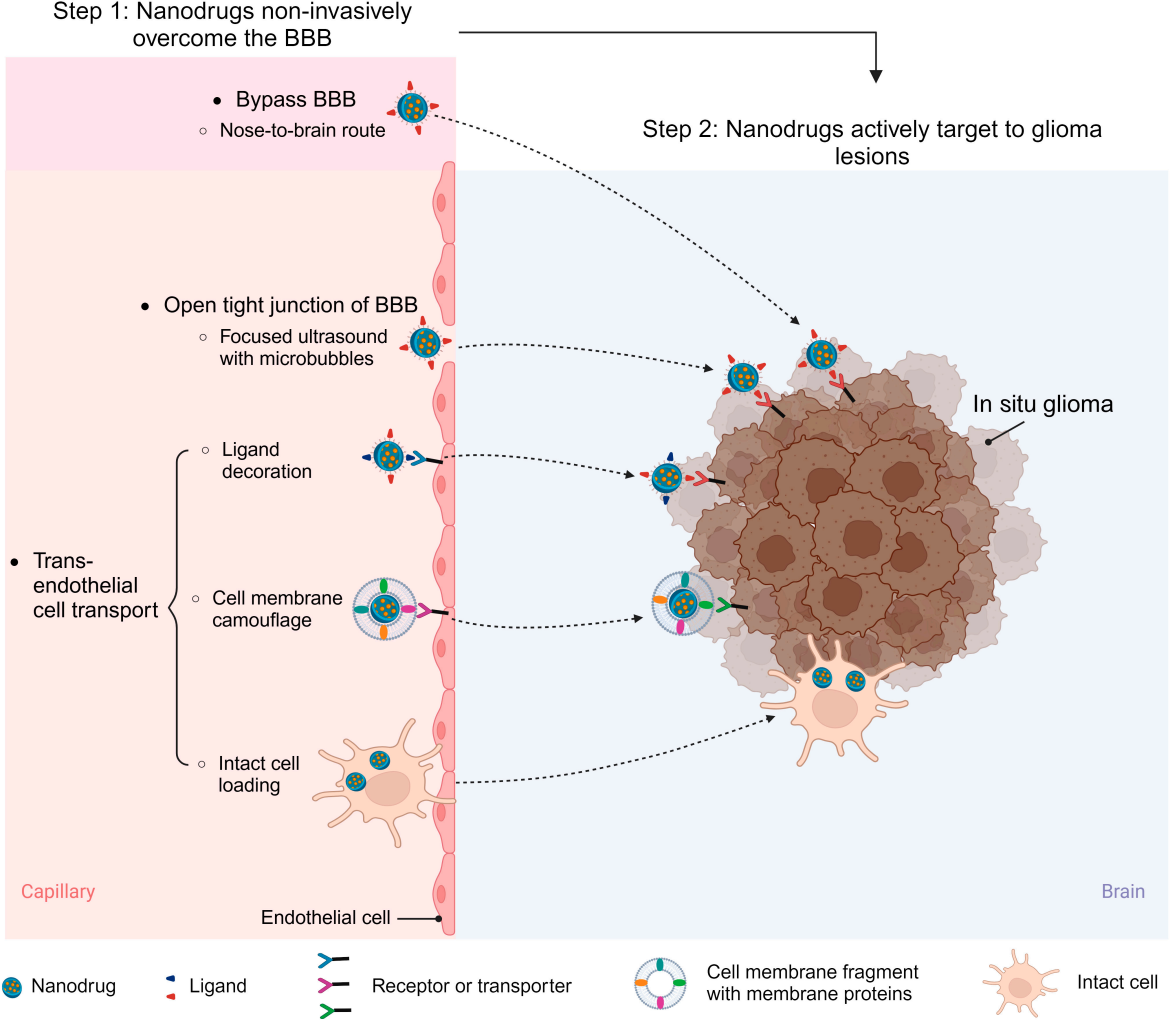
The combined strategies for nanodrug noninvasively overcoming the BBB by bypassing the BBB, opening the tight junction of the BBB or transendothelial cell transport (Step 1), and actively targeting glioma lesions by decoration of nanodrugs using glioma lesion-specific targeted ligands, cell membrane fragments, or intact cells (Step 2) (BioRender).

In terms of overcoming the BBB, unlike invasive methods like direct brain injection that carries high risks, several noninvasive strategies ensure higher safety while overcoming the BBB (Fig. 1, Step 1). The noninvasive ways for nanodrugs to overcome the BBB can be divided into 3 main types: bypassing the BBB, opening a tight junction of the BBB, and transendothelial cell transport. The nose-to-brain pathway is the most common noninvasive method that allows nanodrugs to passively bypass the BBB. Some physical methods like focused ultrasound (FUS) can transiently, reversibly, and locally open the BBB, hence facilitating the passive passage of nanodrugs. Active transendothelial cell transport is usually achieved by decorating nanodrugs using targeted ligands or cell membrane fragments. These decorated strategies aid in the specific recognition of receptors or transporters highly expressed on the cell membrane of BVECs and hence achieving active transendothelial cell transport of nanodrugs. Moreover, intact cells such as immune cells can be utilized to load nanodrugs and assist in crossing the BBB due to their innate abilities [14]. To ensure precise glioma killing with high regard for safety, once the BBB is overcome, nanodrugs need to actively accumulate in glioma lesions (Fig. 1, Step 2). This aim can be typically achieved through the decoration of nanodrugs using glioma lesion-specific targeted ligands, cell membrane fragments, or intact cells. In this review, we will systematically introduce in detail the combined strategies of nanodrugs simultaneously overcoming the BBB and actively targeting glioma lesions for enhancing glioma therapy efficacy.

## The Combined Strategies for Nanodrug Passively Passing the BBB and Actively Targeting Glioma Lesions

### Nanodrugs bypassing the BBB via the nose-to-brain route + actively targeting glioma lesions

Among the noninvasive approaches for nanodrug delivery that effectively bypass the BBB, the nose-to-brain pathway stands out as the most prevalent [15]. This direct route offers a highly patient-friendly and noninvasive alternative, allowing for the efficient transportation of nanodrugs directly to the brain without traversing the BBB, thereby enhancing therapeutic efficacy [16]. The nasal mucosa is rich in capillaries and lymphatic vessels, and nanodrugs can be quickly absorbed through the nasal mucosa into the blood circulation. There is a direct neural connection between the nasal mucosa and the brain, and the nanodrug can directly reach the CNS through the olfactory nerve or trigeminal nerve, which makes nasal administration a unique advantage in the treatment of neurological diseases while minimizing peripheral exposure. Upon entry into the olfactory or respiratory epithelium, nanodrugs traverse the perivascular space or olfactory/trigeminal tract, accessing the cerebrospinal fluid and brain tissue through the perineuronal space [17]. Compared to traditional oral or injectable administration, nasal administration can penetrate the BBB more effectively and deliver the nanodrug directly to the brain, thereby improving treatment effectiveness and reducing side effects. Recent advancements have focused on combining nose-to-brain delivery with targeting-modified nanodrug delivery systems, presenting a precise approach for glioma therapy. After nanodrugs enter the brain via the olfactory or trigeminal nerves, targeting-decorated nanodrugs can selectively identify and penetrate glioma tissue, facilitating effective glioma treatment [18].

**Fig. 2.**
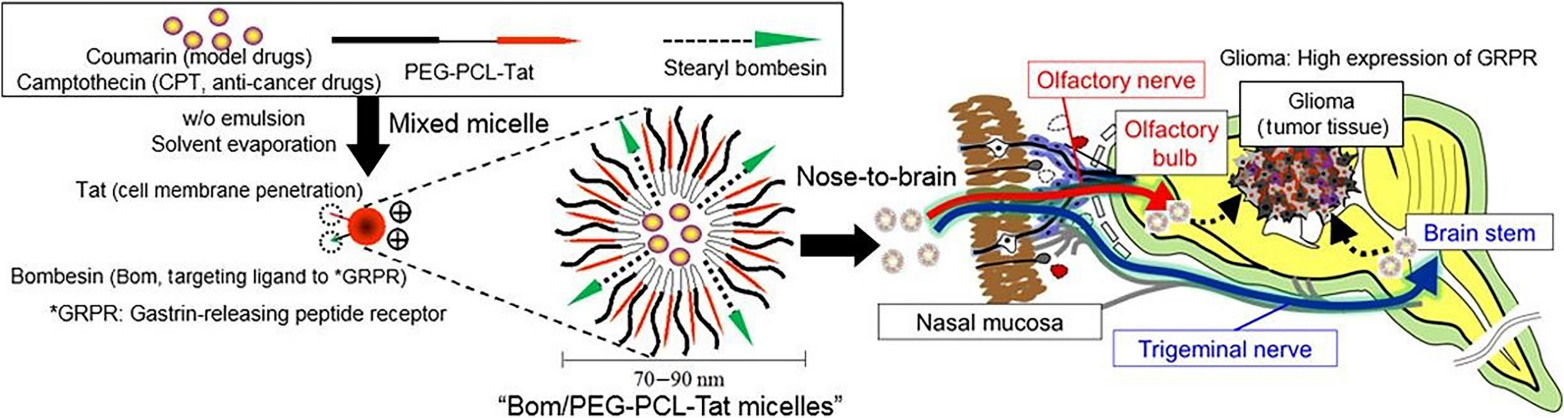
The scheme of preparation of Bom/PEG-PCL-Tat and its delivery process by nose-to-brain and targeted combination. Reproduced from [20] with permission from Elsevier, Copyright 2020.

Many combined strategies have been tried. Chu et al. [19] developed Ephrin type-A receptor 3 (EPHA3) tyrosine kinase antibody-decorated, temozolomide butyl ester (TBE)-loaded polylactide-co-glycolide (PLGA) nanoparticles (NPs) to treat GBM via the nose-to-brain route. EPHA3, a membrane-associated receptor, exhibits notable overexpression in the stroma and vasculature of gliomas while maintaining low expression levels in normal tissues. This characteristic positions it as a promising functional target for GBM treatment. Research findings demonstrate a substantial improvement in the C6 cellular uptake of anti-EPHA3-decorated NPs compared to nontargeting NPs, highlighting its potential for targeted therapy. In vivo biodistribution studies in C6 glioma-bearing mice revealed that anti-EPHA3-decorated NPs had robust fluorescence in the tumor tissues. Anti-EPHA3 demonstrated significant accumulation within the glioma, indicating its specificity. Furthermore, in vivo anti-glioma experiments with C6 glioma-bearing rats demonstrated that TBE-loaded anti-EPHA3-decorated NPs induced markedly higher tumor cell apoptosis and prolonged the median survival time compared to controls. These findings underscore the potent synergy between the nose-to-brain delivery approach and anti-EPHA3-modified NPs for glioma treatment. Kanazawa et al. developed an optimized nose-to-brain nanodrug delivery system, termed Bom/PEG-PCL-Tat. This innovative system comprises poly(ethylene glycol)-block-poly(caprolactone) (PEG-PCL) conjugated with 2 essential peptides: Tat, facilitating cellular penetration, and bombesin (Bom), ensuring precise homing to target cells (Fig. 2) [20]. Bom can specifically target the gastrin-releasing peptide receptor (GRPR). GRPR expression is notably higher on the cell membranes of glioma cells compared to healthy brain cells. Experimental findings demonstrate that Bom/PEG-PCL-Tat micelles exhibit a high uptake rate by GRPR-positive C6 glioma cells while showing no significant increase in uptake by GRPR-negative COS7 cells. Moreover, NPs loaded with camptothecin (CPT) and modified with bombesin demonstrate markedly enhanced cytotoxicity against C6 glioma cells compared to NPs lacking the bombesin modification. Additionally, in an orthotopic C6 glioma rat model, CPT-loaded Bom/PEG-PCL-Tat NPs exhibit superior accumulation in orthotopic gliomas and demonstrate stronger anti-glioma efficacy following nasal administration compared to controls.

### Opening BBB by FUS with microbubbles + nanodrugs actively targeting glioma lesions

The temporary and reversible opening of the BBB is a useful strategy for facilitating the passage of nanodrugs in glioma treatment [21]. Locally opening the BBB via a physical approach is safer compared to chemical disruption, which is typically nonselective and nonlocal, leading to uncontrollable flow of neurotoxic blood components (such as albumin) into the entire brain region, causing edema, neurotoxicity, seizures, aphasia, hemiplegia, and other adverse effects. While various strategies like FUS-mediated microbubble (MB) oscillation, electro-hyperthermia, magnetic hyperthermia, laser, photothermal ablation, transcranial direct current stimulation, and vasodilators for opening the BBB have been developed, there remains a lack of research into the effective combination strategies of locally BBB opening with nanodrugs actively targeting glioma lesions. This gap exists primarily due to the dual nature of BBB opening. While local opening enables nanodrugs to enter the brain parenchyma and exert their therapeutic effects, it also poses risks. An immediate breach in the BBB could potentially allow the infiltration of various macromolecules and harmful agents into the CNS, leading to neuropathological changes and functional impairments.

FUS with gaseous MBs presents a clinically valuable noninvasive physical approach for opening the BBB [22]. MBs, comprising small gas-filled spheres, possess a shell capable of interacting with ultrasound waves. By harnessing momentum transfer from sound waves, ultrasonic fields induce various mechanical phenomena such as acoustic radiation force, microstreaming, and shear stress. MBs are capable of cavitation under ultrasonic irradiation, and this unique interaction obviously diminishes the acoustic energy needed for BBB opening through FUS, consequently bolstering the safety profile of FUS-mediated BBB opening [23,24]. To initiate BBB opening, MBs are injected into the bloodstream and FUS is directed externally to a designated area on the skull using a specialized device. This interaction induces oscillations or vibrations in the MBs, generating mechanical forces such as microstreaming and microjetting [25]. The oscillating MBs exert mechanical effects, inducing localized pressure and mechanotransduction interaction with vascular cells, and further disrupt tight junctions between endothelial cells forming the BBB [26,27]. This disruption precisely opens the BBB at targeted sites, affecting only endothelial cells in direct contact or proximity with activated MBs [28]. Both preclinical and clinical trials employing FUS in conjunction with MBs have validated the safety and efficacy of FUS-created BBB opening in precise drug delivery. This underscores the importance of maintaining precise control over acoustic irradiation parameters and MB concentrations [29]. Combining FUS with MBs demonstrates remarkable efficacy in locally and reversibly opening the BBB with exceptional safety. This approach presents an encouraging opportunity for improving nanodrug delivery into the brain parenchyma. Once the BBB is permeated successfully, nanodrugs can undergo further optimization through targeting modifications, enabling precise and active targeting of glioma lesions (Fig. S1). This strategy holds remarkable potential for improving and targeting tumor therapy effectively.

Numerous integrated approaches employing FUS combined with MBs to facilitate the opening of the BBB and to actively target glioma lesions for the delivery of nanoscale therapeutic agents have been explored. Zhao et al. [30] developed a novel liposome nanomaterial (MB-shBirc5-lipo-NGR) to achieve precise gene delivery in conjunction with FUS. The complex incorporates the NGR peptide decoration, which binds to the CD13 receptor overexpressed in glioma cells and neovascular endothelial cells. Experimental results demonstrated that under the help of FUS, MB-shBirc5-lipo-NGR effectively breached the BBB, specifically targeted C6 glioma cells through NGR/CD13 interaction, and ultimately inhibited glioma growth in an orthotopic glioma rat model. The median survival time of FUS-aided MB-shBirc5-lipo-NGR groups was 38 days, which is extended compared to other groups. Yang et al. [31] devised a multifaceted approach to enhance the efficacy of CRISPR/Cas9-based therapy targeting the O6-methylguanine-DNA methyltransferase (MGMT) gene, a key player in temozolomide (TMZ) resistance in GBM. They engineered lipid–polymer hybrid NPs (LPHNs-cRGD) as carriers for CRISPR/Cas9 plasmids, specifically tailored to target MGMT. To overcome the BBB, they employed FUS in conjunction with MBs to noninvasively and locally disrupt the BBB. Additionally, the surface modification of LPHNs with cRGD ligands improved the accumulation of the CRISPR/Cas9-loaded nanodrugs in GBM lesions, enhancing their therapeutic potential. The synergistic application of FUS and MBs in combination with the LPHNspCas9/MGMT-cRGD NPs led to a significant improvement in the sensitivity of GBM cells to TMZ, both in vitro and in vivo. This innovative strategy holds promise for overcoming drug resistance in GBM and improving the efficacy of CRISPR/Cas9-based therapies in cancer treatment. Yang et al. demonstrated synergistic efficacy in an in situ glioma model in NOD-scid mice, utilizing pulsed high-intensity focused ultrasound (HIFU) combined with phospholipid-coated MBs, and human atherosclerotic plaque-specific peptide-1 (AP-1)-linked liposomes loading doxorubicin (AP-1 Lipo-Dox). This innovative approach was effective against human GBM cells [32]. Initially, pulsed HIFU facilitates the opening of the BBB, enabling enhanced penetration of nanodrugs into the brain parenchyma. This augmented delivery mechanism is further amplified by AP-1 decoration, promoting increased accumulation of AP-1 Lipo-Dox within GBM in comparison with unadorned Lipo-Dox. This enhancement is primarily attributed to the up-regulation of interleukin-4 receptors (IL-4R) on the surface of brain tumor cells. Notably, in vivo treatment experiments demonstrate that the survival time was markedly extended in GBM-afflicted mice subjected to AP-1 Lipo-Dox in conjunction with pulsed HIFU, as opposed to controls (Fig. S2).

## The Combined Strategies for Nanodrug Active Transendothelial Cell Transport and Actively Targeting Glioma Lesions

Implementing a dual-targeting strategy with modified nanodrugs represents a highly effective approach for achieving precise glioma therapy. These nanodrugs not only shuttle across the endothelial cells of BBB but also exhibit selective recognition and penetration of glioma lesions. This dual capability ensures enhanced therapeutic efficacy by precisely targeting and treating the affected areas [33]. Surface modification with ligand, biomimetic camouflage, and intact cell loading are common decorations that help nanodrugs perform dual-targeting functions. Dual-targeting systems are particularly valuable in glioma therapy, where they can help increase the delivery of anticancer medicines to tumor lesions while minimizing damage to healthy tissues. When dual targeting is completed by a single decoration, this system is named the “single decoration-mediated dual-targeting” system, which stands for the easiest method for secondary targeted delivery [34]. When dual targeting is completed by 2 kinds of decorations, this system is defined as the “2 decoration-mediated dual-targeting” system. Further, when triple targeting is completed by single or 2 kinds of decorations, their systems can be named separately as “single decoration-mediated triple-targeting” system or “2 decoration-mediated triple-targeting” system. The blood–brain tumor barrier (BBTB) is often used as a third target in glioma therapy. The detailed applications of these multitargeted nanodrug systems in glioma treatment are outlined as follows:

### Single decoration-mediated dual-targeting system

In this system, through a single modification technique, nanodrugs can simultaneously achieve the crossing of BBB by transendothelial cell transport and the precise targeting of glioma lesions. Currently, there are mainly 3 methods to achieve this dual-targeting function: first, loading nanodrugs into intact cells that can cross the BBB and actively target gliomas; second, modifying single ligand on the surface of nanodrugs, and the ligand has the functions of both BBB targeting and glioma targeting; third, disguising nanodrugs with a single type of cell membrane fragments that have both BBB targeting and glioma targeting capabilities to achieve a dual-targeting effect. These methods not only simplify the modification process but also improve the therapeutic efficiency and accuracy of drugs.

#### Intact cell-loaded nanodrugs

Immune cells and platelets are the most commonly used cells for loading nanomedicines because of their inherent capacity to migrate from the bloodstream to brain tumor tissues, which renders them naturally superior vehicles for delivering nanodrugs to traverse the BBB and particularly accumulation in glioma lesions [35–37]. The common immune cells and platelets for nanodrug delivery and their detailed application in glioma therapy are introduced below.

##### Neutrophils

Neutrophils (NEs) exhibit remarkable mobility and chemotaxis, enabling them to swiftly respond to infection or inflammatory cues like tumors. Specific factors within the glioma microenvironment, notably tumor necrosis factor-α, and ceruloplasmin, actively attract and stimulate neutrophils to migrate toward and infiltrate glioma tissues [38]. Zhang’s team utilize NEs to carry paclitaxel-loaded liposomes (PTX-CL) to treat residual glioma tissues after surgery [36]. In vitro BBB and 3D tumor spheroid models showed that inflammation primed PTX-CL/NEs successfully across BBB and penetrated glioma, respectively. In vivo results revealed that PTX-CL/NEs not only effectively retarded the recurrent glioma, but also notably enhanced the survival rates of mice who had undergone glioma surgery. Furthermore, these findings demonstrated that inflammatory factors released following glioma resection directed movement of PTX-CL/NEs to reach glioma tissues and then PTX release to eliminate residual glioma cells. Ding et al. presented an innovative semiconducting polymer nano-therapeutic system, SPC_Fe_/siP, designed to facilitate efficient delivery to orthotopic glioma sites. SPC_Fe_/siP comprises a semiconducting polymer, specifically engineered to encapsulate programmed death-ligand 1 siRNA and iron oxide (Fe_3_O_4_) NPs within a singlet oxygen (^1^O_2_)-responsive nanocarrier. The surface of this nanocarrier is decorated with sialic acid, a ligand that selectively targets neutrophils. This targeted interaction enables SPC_Fe_/siP to effectively bind to neutrophils, transforming them into Trojan horses that traverse the BBB and enhance delivery to glioma sites. Finally, this neutrophil–nanodrug conjunction system achieved sono-activatable ferroptosis immunotherapy via in vitro and in vivo studies [39].

##### Macrophages

**Fig. 3.**
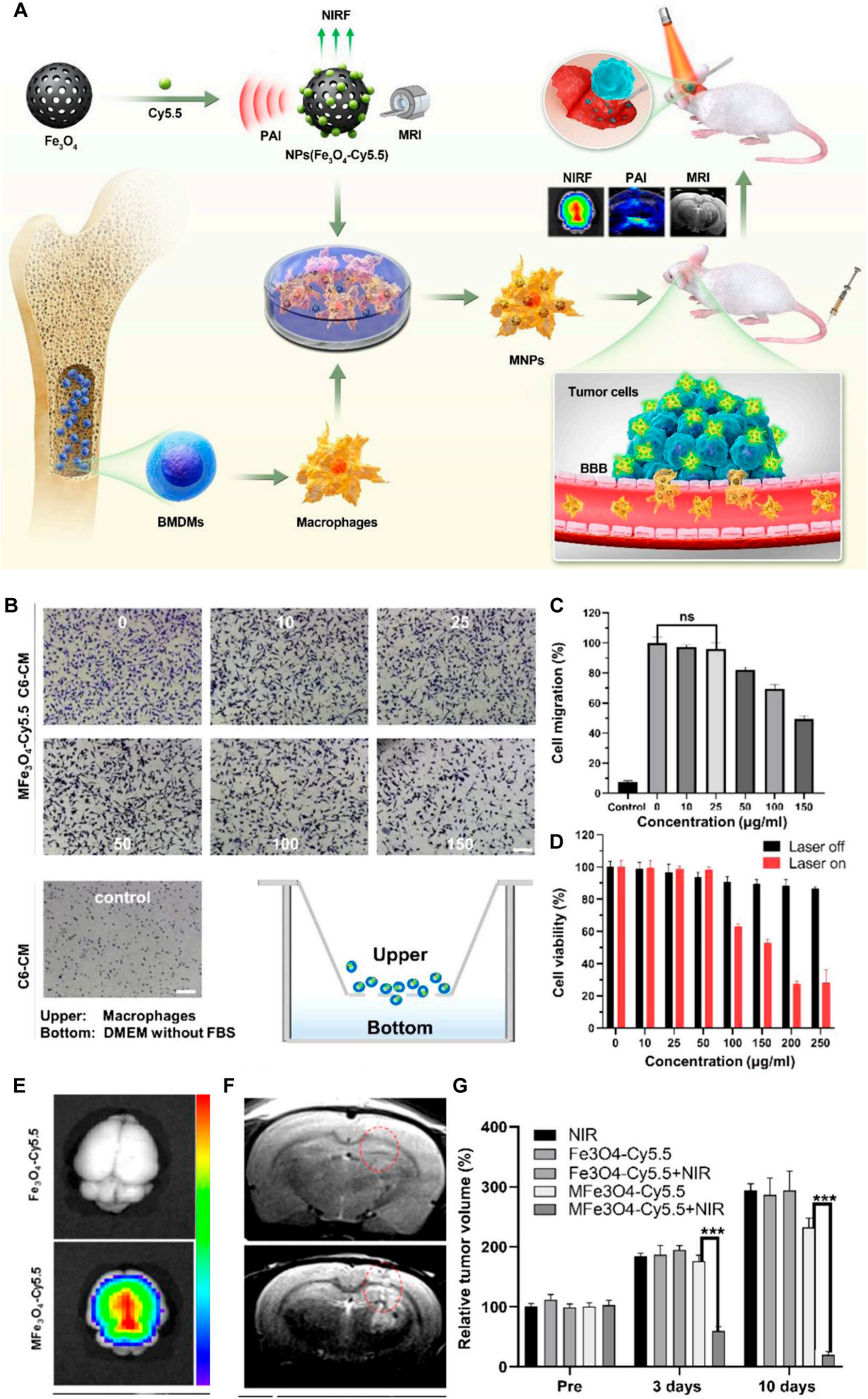
(A) Scheme of the synthesis process and application of MFe_3_O_4_-Cy5.5. (B) The migration illustration of macrophage in vitro. (C) Migration ratio of macrophages with various treatments. (D) Cell viability with and without NIR irradiation under treatment with different Fe_3_O_4_-Cy5.5 concentrations. (E) Optical imaging and (F) magnetic resonance imaging of Fe_3_O_4_-Cy5.5 and MFe_3_O_4_-Cy5.5 in the brain with tumor. (G) Quantitative brain tumor volume of mice with different treatments. Reproduced from [41] with permission from the American Chemical Society, Copyright 2021.

In neuropathological diseases like glioma, extra-peripheral macrophages can be engaged from the bone marrow to the brain. Recruitment factors are usually from glioma cells themselves [40]. Therefore, tumor-associated macrophages are also frequently utilized for nanodrug delivery in glioma treatment. Typically, a macrophage-loaded photothermal nanoprobe (MFe_3_O_4_-Cy5.5) was employed in glioma therapy [41]. In vitro migrated experiment indicated that MFe_3_O_4_-Cy5.5 possesses the ability to migrate toward C6 glioma cells. In vitro BBB model and in vivo imaging illustrated that MFe_3_O_4_-Cy5.5 could cross the BBB and accumulate within glioma tissues. Moreover, once MFe_3_O_4_-Cy5.5 enters glioma lesions, it is capable of performing multimodal imaging, guiding glioma surgery, and suppressing glioma growth through photothermal therapy under near-infrared (NIR) light irradiation, thereby enhancing the survival rate of glioma-bearing mice (Fig. 3). Ibarra et al. [42] used macrophages to carry and deliver conjugated polymer NPs (CPNs) to improve photodynamic therapy (PDT) in GBM. Macrophages loaded with CPNs maintain their natural activity and function, enabling them to target and penetrate GBM spheroids and the orthotopic GL261 GBM model. In an in vitro 3D GBM model, PDT efficacy was significantly enhanced when macrophages were used as a delivery vehicle for CPNs, compared to non-vehicle CPNs.

##### Dendritic cells

The inherent migration ability of dendritic cells (DCs) reaching the glioma area and maintaining the immune response of glioma tissues makes them promising nanodrug delivery agents for glioma treatment [43]. Li et al. [44] employed DC to carry the doxorubicin–polyglycerol–nanodiamond compound (Nano-Dox) to improve the antigenicity of GBM cells and trigger an anti-GBM immune response. After intravenous injection of Nano-Dox-loaded mouse bone marrow-derived DCs (mDCs) into GBM-bearing mice, the Nano-Dox-loaded mDCs can cross the BBB and specifically target GBM lesions. The Nano-Dox-treated GBM cells exhibited not only abundant damage associated with molecular pattern secretion but also antigen release, hence inducing immunosuppression to GBM cells.

##### Platelets

Studies have confirmed that platelets have a variety of functions such as vascular endothelial adhesion, tumor-targeting, secretion of intracellular substances after activation, and immune evasion functions [37], making them promising for camouflaging nanodrugs in antitumor therapy. Jiang et al. [45] loaded Dox-carrying, NIR light-responsive nanomedicines, and magnetic NPs into platelets, naming the complex FeDN@P. In vivo orthotopic glioma model studies demonstrated that this NP/platelet complex can accumulate in the brain under the influence of a magnetic field, and then cross the BBB via the platelet’s vascular adhesion ability, migrating into the brain parenchyma. Leveraging the tumor cells’ ability to induce platelet aggregation, FeDN@P can specifically accumulate in glioma tissues. Upon platelet activation, the DPT NPs within were effectively released and entered glioma cells. Subsequently, under the dual action of reactive oxygen species and laser irradiation, the structure of the DPT NPs was disrupted, releasing Dox to exert its tumor-suppressing effects. Finally, the combination of photothermal therapy and chemotherapy effectively killed glioma cells, inhibited angiogenesis, enhanced immune response, and prolonged the survival cycle of glioma-bearing mice in situ.

#### Single ligand decorated nanodrugs

Given the high expression of numerous receptors or transporters simultaneously on both BVEC membranes and glioma cell membranes (GCMs), there exists a unique opportunity for a dual-targeting strategy utilizing a single ligand to effectively pass through the BBB and target gliomas concurrently. As reported, the highly expressed receptors on both BVEC membranes and GCMs include the low-density lipoprotein receptor (LDLR) family [46], transferrin receptor (TfR) [47,48], lactoferrin receptor (LfR) [49], folate receptor (FR) [50], insulin receptor (IR) [51], neuropeptide Y (NPY) Y1 receptor (Y1R) [52], nicotinic acetylcholine receptors (nAChRs) [53], and interleukin-6 receptor (IL-6R) [54]. Highly expressed transporters on both BVEC membranes and GCMs include glucose transporter (GLUT1) [55], large neutral amino acid transporter (LAT1) [56,57], and organic cation/carnitine transporter 2 (OCTN2) [58,59]. The commonly used types of targeted ligands include peptides, aptamers, antibodies, antibody fragments, proteins, and small molecules [60–63]. The summarized application of the “one ligand-mediated dual-targeting” strategy in glioma treatment is shown in Table, and the most relevant receptors and transporters are introduced in detail below.

**Table.** The utilization of nanodrugs featuring dual-targeting functionality mediated by a single ligand holds promising applications in the treatment of glioma. The full names of the various types of “therapeutic strategy” are as follows: CT, chemotherapy; IT, immunotherapy; GT, gene therapy; TT, targeted therapy; PTT, photothermal therapy.

Targets	Ligands		Nanocarrier	Used drug “therapeutic strategy”	Cell model	Ref.
	Type	Ligand				
LDLR	Peptide	Peptide-22	PEG-PLA	Paclitaxel “CT”	C6	[64]
		L-4F	Extracellular vesicles	Methotrexate + pro-apoptotic peptide KLA “CT + peptide therapy”	U87	[65]
		mApoE	Liposomes	Doxorubicin “IT”	Patient-derived GSCs	[66]
LRP	Peptide	SRL	Poly(amidoamine)	pEGFP-N1 plasmid “GT”	C6	[67]
		Angiopep-2	PEG-b-P(GuF)/Ang-PEG-b-P(Gu)	CRISPR/Cas9 “GT”	U87MG	[142]
			Fe_3_O_4_@mSiO_2_/exosomes	Brequinar (BQR)/siGPX4/Fe^2+^ “Synergistic ferroptosis”	LN229/A172	[143]
			PEG-b-P(Gu/Hb)/Ang-PEG-b-PGu	Anti-miR-21/miR-124 mimic “TT”	U87MG	[72]
		RAP12	PEG-PLA	Paclitaxel “CT”	U87	[68]
		ST-RAP12	PEG-PLA	Paclitaxel “CT”	U87	[69]
LRP1, LRP2, LDLR	Peptide	ApoE	Pullulan/poly(deca-4,6-diynedioic acid)	Temozolomide (TMZ)/indocyanine green (ICG) “CT/NIR irradiation”	U87MG/CSC2	[46]
			Dextran	ABT-263 & A-1210477 “CT”	U87MG/U251	[70]
TfR	Peptide	T7	Exosome	Galectin-9 siRNA “TT”	GL261	[144]
		T12	PEG-PLA	Paclitaxel “CT”	U87MG	[74]
		B6	Liposome	Vincristine (VCR) “CT”	GL261	[145]
		CRT	PEG-PLGA	Paclitaxel “CT”	C6	[146]
	Antibody fragment	TfRscFv	Liposome	p53 plasmid “TT”	U87	[147]
	Antibody	OX26	PEG-*b*-PLA/PEG-*b*-P(LA-co-DHC)	RhB “imaging”	C6/L929	[76]
		RI7217	Liposome	Docetaxel “CT”	U87-MG	[148]
	Protein	Tf	PEGylated nanoscaled graphene oxide	Docetaxel “CT”	C6	[77]
LfR	Protein	Lactoferrin	BSA	Doxorubicin “CT”	C6	[149]
			Graphene oxide@Fe_3_O_4_	Doxorubicin “CT”	C6	[150]
Y1R	Peptide	AP-NPY	DSPE-PEG	Doxorubicin “CT”	U87MG	[52]
		^D^APT	DSPE-PEG	IRDye780 “PTT and photoacoustic imaging”	U87-MG	[151]
FR	Small molecular	Folate	Carbon nanosphere	Doxorubicin “CT+IT”	GL261	[152]
			mPEG-PCL	Pterostilbene (Pt)	A172	[80]
nAChR	Peptide	^D^CDX	liposome	Doxorubicin “CT”	U87	[81]
		RVG29	Zein-derived NPs	Dactolisib (Dac) “TT”	U87	[82]
		D8	Liposome	Doxorubicin “CT”	U87	[126]
IL-6R	Peptide	I_6_P_8_	PEG-PLGA	Doxorubicin “CT”	U251	[54]
GLUT1	Small molecular	Glucose	BBR-Glu nanoparticles	Berberine “TT”	U251/U87	[84]
		Glucosamine (G)	Silicon	Indocyanine green (ICG) “PTT”	U87	[153]
		2-Deoxy-d-glucose	Poly(ethylene glycol)-b-poly(trimethylene carbonate)	Paclitaxel “CT”	RG-2	[83]
		1,2-O-isopropylidene-α-D-glucofuranose	Poly(ethylene glycol)-poly(L-glutamic acid)	Cisplatin “CT”	U87MG	[154]
LAT1	Small molecular	Glutamate	Liposomes	Docetaxel “CT”	C6	[85]
		Tyrosine	Polyethylene glycol stearate	TMZ and sorafenib “CT+TT”	U87MG	[57]
		Amphi-DOPA	Liposomes	WP1066 “TT”	GL261	[155]
OCTN2	Small molecular	L-carnitine	PEG-PLGA	Paclitaxel “CT”	T98G	[59]

##### Targeting the LDLR family

The LDLR family comprises some cell surface endocytic receptors that bind to and internalize extracellular ligands. The members of LDLR family include LDLR, LDLR-related protein 1 (LRP1), and LDLR-related protein 2 (LRP2). Thus far, common ligands decorated on the surface of nanodrugs that target the LDLR family are mainly various artificially synthetic peptides. Peptide-22 [64], L-4F [65], and mApoE [66] are usually used to target LDLR; Angiopep-2 [33], SRL [67], RAP12 [68], and stapled RAP12 (ST-RAP12) [69] are often used to target LRP; Apolipoprotein E (ApoE) [70] is used to simultaneously target LDLR, LRP1, and LRP2. Zhang et al. [64] constructed peptide-22 decoration and PTX-loaded NPs (PNP-PTX). Their findings revealed that peptide-22 decoration remarkably enhanced the cellular uptake of PNP by C6 glioma cells and BCECs in vitro, while extra peptide-22 hindered this uptake. In vivo and ex vivo fluorescence imaging demonstrated that peptide-22-modified PNP could effectively cross the BBB and exhibited greater accumulation in glioma tissues compared to unmodified NPs. Additionally, in vivo glioma treatment illustrated that peptide-22-modified PNP-PTX markedly lengthened the median survival time of mice with orthotopic glioma in comparison with other treatment groups. In our prior research, we synthesized Angiopep-2 decorated high-molecular polymers that can load siRNAs [71] or miRNAs [72] by triple interactions (electrostatic, hydrogen bonding, and hydrophobic) to form nanomedicines. Experiments showed that these LRP1-targeted nanomedicines demonstrated exceptional BBB penetration in vitro and robust tumor accumulation in vivo. Furthermore, by employing Angiopep-2-modified nanomedicine, incorporating dual-siRNA targeting polo-like kinase 1 and vascular endothelial growth factor receptor-2, or dual-miRNA targeting miR-21 and miR-124, we observed significant inhibition of orthotopic U87MG tumor growth, along with a notable extension in the middle lifespan of the murine model. To further improve targeting efficiency, Jiang et al. [73] applied ApoE to modify nanodrugs, and results showed that ApoE enhanced chimeric polymersome (CP) penetration through bEnd.3 monolayers by 2.2-fold compared to Angiopep-2, as evidenced by BBB model experiments. Moreover, the ApoE-installed CP showed active accumulation and efficient penetration in orthotopic U87MG GBM. These phenomena contributed to the triple-targeting ability of ApoE to LDLR, LRP1, and LRP2. Tail vein injection of SAP-loaded ApoE-CP achieved antitumor growth in the U87MG GBM mice model and significantly improved survival rates of mice with orthotopic glioma without observable adverse effects.

##### Targeting TfR

The function of TfR is endocytosis of Tfs, which serves as a hydrophilic carrier of iron ions in the bloodstream. Since TfR is overexpressed in both the BBB and glioma cells, but less so in noncancerous cells, it stands out as a highly promising target site in anti-glioma treatment [74]. Nowadays, various ligands such as peptides, antibodies, antibody fragments, and intact proteins have been effectively employed to functionalize nanodrugs, facilitating targeted delivery to both the BBB and glioma lesions via binding to the TfR. T7 peptide can specially bind to the TfR. Liu et al. [75] constructed a T7 peptide-modified exosome (T7-exo). Results exhibited that the T7-exo can not only efficiently encapsulate and shield cholesterol-modified Cy3-siYY1, but also facilitate quick payload release in a cytoplasmic reductive condition. In vitro and in vivo experiments illustrated the engineered T7-siYY1-exo excellent BBB-crossing and GBM targeting efficiency, which hence promote superior drug delivery in GBM. Importantly, in vitro studies revealed that T7-siYY1-exo not only potentiated the responsiveness to chemoradiotherapy but also overcame resistance. Furthermore, the combination of T7-siYY1-exo with TMZ/IR exhibits a cooperative antitumor result against GBM, making a substantial improvement in the survival rate of GBM-bearing mice. Yue et al. [76] engineered RhB-loaded micelles employing mal-PEG-b-PLA and mPEG-b-P(LA-co-DHC/RhB) block copolymers, with the surface adorned by a targeted anti-TfR antibody, OX26. In vitro assessments revealed that the attachment of OX26 significantly increased the C6 cellular uptake to micelles. Furthermore, pharmacokinetic and biodistribution experiments illustrated the ability of OX26-decorated micelles to cross the BBB and selectively target glioma lesions. Liu et al. [77] developed Tf-linked PEGylated nano-structured graphene oxide (GO) packing Dox, termed Tf-PEG-GO-Dox. In vitro experiments demonstrated that Tf-modified nanodrug can be more uptaken, thus resulting in the stronger killing of C6 glioma cells compared to non-Tf-conjugated nanodrug and free Dox. Furthermore, in brain glioma-bearing rats, Tf-modified nanodrug displayed the most potent antitumor effect among the tested formulations. Therefore, these findings suggested that Tf facilitated Dox transport across the BBB to reach gliomas.

##### Targeting FR

FR, a glycosylphosphatidylinositol (glycolipid)-associated receptor, is usually highly expressed on the cell membrane of glioma cells and BEVCs [50]. FR primarily facilitates its endocytosis by binding with high affinity to folate and reduced folic acid (FA) derivatives [78]. Therefore, low-molecular-weight folate, commonly known as FA, serves as a highly effective ligand for targeting folate receptors in a nanodrug delivery system. Afzalipour et al. [79] developed FA-conjugated magnetic NPs (MNPs) coated with a triblock polymer (PEG/PBA/PEG) and loaded with TMZ. The TMZ/MNPs-FA demonstrated effective crossing of the BBB and selective targeting of C6 glioma cells in both in vitro and in vivo studies. Additionally, when exposed to a magnetic field, TMZ/MNPs-FA exhibited dual targeting and superior therapeutic efficacy, resulting in substantial inhibition of glioma growth, increased rat body weight, and prolonged survival compared to control groups. Wang et al. [80] successfully contributed the pterostilbene (Pt)-loaded and folate-decorated polymeric micelle, named F-Pt/M. Compared to the nontargeted NP, F-Pt/M exhibited a substantially greater accumulation in GBM cells via FR-mediated endocytosis. Therefore, F-Pt/M showed higher cytotoxicity against GBM cells. Furthermore, this optimized F-Pt/M system significantly improved the ratio of Pt crossing the BBB through receptor-mediated endocytosis in brain distribution study in vivo.

##### Targeting nAChR

The nAChRs are receptor polypeptides that produce a response to the neurotransmitter acetylcholine and are often overexpressed on both cell membranes of BVECs and glioma cells [53]. The D-peptide ligand of nAChRs (^D^CDX), a 29-mer peptide derived from Rabies virus glycoprotein (RVG29), and D8 peptide are the main ligands used for targeting nAChRs in the nanodrug delivery system. Wei et al. [81] harnessed ^D^CDX modification to improve the delivery of Dox via liposomes. The in vitro experiments demonstrated that this modification notably promoted liposomes across the BBB and targeted glioma cells, thereby enhancing the therapeutic efficacy of Dox for GBM cells. Zhang et al. [82] created zein-based NPs linked with RVG29 and encapsulated with Dactolisib (Dac), termed zein-RVG-Dac_NP, to treat GBM. In vivo experiments demonstrated that tail vein injection of zein-RVG-Dac_NP led to a significant increase in Dac accumulation within orthotopic brain tumors in mice, effectively suppressing tumor growth. This outcome highlights the nanodrug’s ability to cross the BBB and selectively accumulate in GBM regions using nAChR-mediated pathways. Subsequent release of Dac within GBM cells further contributed to their inhibition.

##### Targeting GLUT1

GLUT1, overexpressed on both BVECs and glioma cells, serves as the most efficient transporter for rapid glucose uptake, crucial for fueling glioma growth. Glucose, glucosamine, 2-deoxy-D-glucose, and 1,2-O-isopropylidene-α-D-glucofuranose are common small molecular ligands targeting GLUT1. Jian et al. [83] devised dual-targeted functionality by developing NPs (dGlu-NP) using 2-deoxy-D-glucose-modified poly(ethylene glycol)-co-poly(trimethylene carbonate). In vitro studies revealed that RG-2 glioma cells internalized a higher amount of dGlu-NP compared to nonglucosylated NPs (NP/PTX). Furthermore, dGlu-NP exhibited increased permeation across the BBB. Notably, PTX-loaded dGlu-NP (dGlu-NP/PTX) exhibited greater cytotoxicity toward RG-2 cells than NP/PTX. In vivo fluorescent imaging illustrated that compared to other controls, more dGlu-NP accumulated at glioma sites due to its dual-targeting capability. Additionally, the anti-glioblastoma effectiveness of dGlu-NP/PTX was remarkably improved in comparison to Taxol and NP/PTX. Wang et al. [84] successfully crafted glucose-modified berberine (BBR-Glu) NPs that exhibit enhanced penetration into glioma cells (U87 and U251), thereby inducing a higher level of cytotoxicity compared to BBR-Water. BBR-Glu demonstrated superior imaging capabilities in both brains and gliomas in mice with orthotopic U87 glioma, suggestive of an enhanced ability of BBR to cross the BBB and specifically target glioma lesions.

##### Targeting LAT1

Various amino acids and amino acid derivatives usually are used for LAT1 targeting. Li et al. [85] built docetaxel-loaded glutamate-D-α-tocopherol polyethylene glycol 1000 succinate copolymer (Glu-TPGS) functionalized LAT1-targeting liposomes (DTX-TGL) to manage glioma killing. In vitro results demonstrated that, in contrast with unmodified liposomes, the DTX-TGL-treated group exhibited significantly higher cellular uptake and cytotoxicity. Furthermore, in vivo fluorescent imaging revealed that TGL exhibited superior BBB penetration and glioma-targeting efficiency in mice compared to its unmodified counterparts. Zhang et al. [57] utilized tyrosine-modified polyethylene glycol stearate to develop LAT1-targeting NPs (L-NPs) that carry both TMZ and sorafenib (L-STNPs). These L-STNPs can traverse the BBB, gather in the U87MG glioma sites, contribute to the binding of tyrosine with LAT1, and effectively combat tumors through apoptosis and ferroptosis, which are mediated by TMZ and sorafenib.

#### Single-type cell membrane camouflaged nanodrugs

Biomimetic modifications to nanodrugs have garnered increasing attention due to their remarkable biocompatibility and inherent functionalities [86]. Employing natural bio-membrane fragments, such as cell membrane fragments, as the encapsulating shell for synthetic NPs is a proven strategy to extend blood circulation time, enhance BBB penetration, and specifically target gliomas with nanodrugs. Thus far, the available cell membrane types that facilitate dual targeting in glioma therapy primarily consist of GCMs, immune cell membranes (ICMs), and platelet membranes (PMs) [87–89]. The summarized application of the “single cell membrane camouflage-mediated dual-targeting” strategy in glioma treatment is shown in Table S2.

##### Glioma cell membrane

The concept of GCM camouflage was inspired by the glioma cells’ ability to easily traverse the BBB by overexpressing intercellular adhesion molecule-1 (ICAM-1) and down-regulating specific proteins in tight junctions [90]. Additionally, these cells localize with homologous cells because of homotypic recognition facilitated via the rich proteins present on the cancer cell membrane [91]. Zou et al. utilized acetalated dextran (Ac-DEX) to physically encapsulate TMZ and CDDP, creating NPs@TMZ+CDDP. Subsequently, they coated the surface of these NPs with GBM cell membranes, resulting in MNPs@TMZ+CDDP [92]. These modified NPs exhibited remarkable capabilities of crossing the BBB and targeting GBM cells. Additionally, TMZ and CDDP were able to be released simultaneously under acidic pH conditions within endo/lysosomes, effectively killing GBM cells. Mice implanted with orthotopic U87MG or TMZ-resistant U251R GBM and treated with MNPs@TMZ+CDDP demonstrated a powerful antitumor effect, significantly prolonging their length of survival compared to mice treated with NPs loaded with a single drug. Zhang et al. [93] utilized GBM patient-derived tumor cell membrane (GBM-PDTCM) to camouflage gold nanorods (AuNRs). Leveraging the remarkable homology of GBM-PDTCM to the brain cell membrane, GBM-PDTCM@AuNRs exhibited remarkable efficiency in traversing the BBB and specifically targeting the GBM. Furthermore, because of the functionalization of Raman reporter and lipophilic fluorophore, GBM-PDTCM@AuNRs are capable of emitting fluorescence and Raman signals at GBM lesions. Guided by these dual signals, surgeons can precisely resect nearly all tumors in a mere 15 minute, thereby enhancing surgical outcomes for advanced GBM. Additionally, intravenous administration of GBM-PDTCM@AuNRs effectively performs PTT in orthotopic xenograft mice, resulting in a doubled median survival time, and obviously improving nonsurgical treatment options for early GBM.

##### Immune cell membrane

Tumorigenesis typically coincides with the infiltration of various immune cells, including neutrophils, macrophages, monocytes, DCs, natural killer cells, and lymphocytes. Those immune cells actively accumulate in tumor areas, making ICMs a highly effective choice for camouflaging NPs and assisting in immune evasion [94,95].

Macrophage membranes are the most widely used in decorating NPs for glioma therapy among immune cells. Macrophages can cross the BBB and accumulate in glioma tissue. One plausible mechanism involves the contact between integrin α_4_/β_1_/αvβ_3_, Macrophage-1 antigen (Mac-1), and CC-chemokine ligand 2 (CCL2) present on the cell membranes of macrophages [96,97] and vascular cell adhesion molecule-1 (VCAM-1) present on the cell membranes of BVECs [98,99] and glioma cells [100]. Lai et al. [101] created macrophage membrane camouflaged DSPE-PEG NPs loading NIR Ib (NIR-Ib) fluorescence dye IR-792, and the NP was named MDINP. MDINPs were capable of traversing the BBB and specifically moving into glioma tissues due to CAM-mediated endocytosis. This process was facilitated by the interaction between surface antibodies like integrin α_4_ on MDINPs and VCAM-1/ICAM-1 receptors on endothelial and GBM cells. Once they reached glioma cells, IR-792-loaded MDINPs functioned as NIR-Ib fluorescence probes achieving targeted GBM imaging. Furthermore, these NPs could eliminate GBM cells through the photothermal effect of NIR-Ib. The NIR-Ib probe-directed PTT effectively prevented GBM development and extended the lifespan of mice with orthotopic GBM. Xiao et al. [102] reported macrophage membrane-camouflaged multifunctional polymer nanogels based on poly(N-vinylcaprolactam) coloaded with manganese dioxide (MnO_2_) and cisplatin to cross the BBB for orthotopic glioma targeting, magnetic resonance (MR) imaging, and combinational chemotherapy/chemodynamic therapy. The decoration of macrophage membranes extended the circulation periods of nanogels in blood and facilitated their traversal of the BBB and active accumulation in glioma cells. This was achieved due to the presence of VCAM-1 in bEnd.3 and glioma cells, which can combine with certain integrins such as α_4_ and β_1_, expressed on the surface of macrophages. Additionally, nanogels were designed to release Mn^2+^ and cisplatin under acidic conditions and high concentrations of GSH (10 mM) within tumor cells. The released Mn^2+^ enhanced chemotherapy and enabled T1-weighted MR imaging. The released cisplatin promoted the formation of H_2_O_2_ for downstream generation of ROS to induce glioma cell death. Notably, the use of macrophage membrane-camouflaged nanogel led to the most significant glioma development inhibition compared to all other groups (Fig. S3).

Apart from macrophage membranes, tumor-antigen-activated DC membranes and natural kill cell membranes were also reported to create excellent camouflage for nanomedicines in glioma treatment. Ma et al. developed an activated mature DC membrane (aDCM)-camouflaged and rapamycin (RAPA)-carried nanodrug, named aDCM@PLGA/RAPA. This nanodrug demonstrated an improved capacity for efficiently crossing the BBB and remarkable homotypic targeting capabilities toward C6 glioma cells in vitro and in vivo [103]. The homotypic targeting abilities of aDCM were attributed to the fact that tumor antigen-activated DCs exhibited higher expression of CD80/86 and MHC I/II molecules on their membrane. Because of the close association between tumor antigens and tumor cell lysate (TCL) throughout the immune response, these tumor antigens can be processed and presented on the DC membranes. Apart from the glioma cell killing efficacy induced by RAPA, aDCM@PLGA/RAPA also elicited an immune response in the glioma area. aDCM is capable of stimulating immature DCs to mature ones, thereby further activating other immune cells, including tumor-infiltrating T cells and natural killer cells. This activation finally triggered more immune responses toward glioma therapy. Deng et al. [104] constructed a natural killer cell simulation nanorobot (NK@AIEdots) with aggregation-induced emission (AIE) properties by camouflaging a natural killer cell membrane on the inner skeleton of AIE-active polymer. In vitro and in vivo detection illustrated that NK cell membrane coating remarkably improved the efficiency of AIEdots crossing the BBB. The basic principle was that the integrins retained in NK cell membranes bind with cell adhesion molecules on the surface of endothelial cells, triggering a signaling delivery inside cells. This cascade damaged tight junctions and reorganized the actin cytoskeleton, resulting in new intercellular gaps at the BBB. The NK@AIEdots also showed strong U87MG glioma-targeting ability in vitro and in vivo because some membrane proteins including NKG2D and DNAM-1 powering tumor recognition ability were kept on NK cell membranes. NKG2D could recognize MHC class I-related molecules and stress-derivable proteins expressed on the surface of U87MG cells. Additionally, DNAM-1 was capable of recognizing PVR and Nectin-2, both being highly expressed on the surface of U87MG cells. After crossing the BBB and accumulating in orthotopic glioma lesions, the NK@AIEdots achieved effective photothermal therapeutic efficacy under 808-nm laser irradiation and significantly inhibited glioma development.

##### Platelet membrane

PMs are also effective tools for camouflaging nanodrugs due to tumor recruitment into platelets. Li et al.[105] employed PMs to camouflage Dox-loaded nanogels (Dox@PNGs). At the cellular level, the results showed that CD62P on the surface of PMs facilitated the targeting of Dox@PNGs to glioma cells by specifically binding to CD44 on glioma cell surfaces. Results from an in vitro BBB model and an in situ glioma model further confirmed that CD62P aids Dox@PNGs in crossing the BBB. Additionally, CD47 on the PM helps Dox@PNGs evade immune attacks by binding specifically to SIRPα on macrophages. Ultimately, with the assistance of PMs, more Dox was delivered into C6 glioma cells, resulting in enhanced tumor cell killing, inhibition of vasculogenic mimicry, and suppression of in situ tumor growth.

### Two decoration-mediated dual-targeting system

Even though single decoration simplifies the design of nanodrugs, it continues to limit targeting efficiency with limited internalization by BVECs and glioma cells [106]. Fortunately, 2 decorations can help resolve the backwardness of a single decoration. According to different aims, the 2 decoration-mediated dual targeting can be divided into 2 types. One type is that 2 kinds of decorations simultaneously have BBB and glioma lesions’ dual-targeting function, which is used to further improve the targeted efficacy. Another type is that one decoration targets the BBB and another decoration targets glioma lesions to satisfy the expressed diversity of receptors or transporters on 2 different targeted sites.

#### Two decorations simultaneously target both the BBB and glioma lesions

##### Two kinds of ligands

Because of the limited targeting efficiency of single modification with Glu or vitamin C (Vc) with a 2- to 4-fold increase compared to nontargeting decoration [107], Peng et al. [108] designed glucose and Vc co-decorated liposome (Glu-Vc-Lip) to deliver PTX for glioma treatment. The result showed that Glu-Vc-Lip had 1.95-fold and 4-fold higher glioma cellular uptake than Glu-Lip and Vc-Lip, respectively. In fluorescence imaging of mice bearing C6 glioma, more Glu-Vc-Lip accumulated at tumor sites than controls. Liu et al. [109] also constructed biotin and glucose co-modified liposomes to deliver PTX owing to more biotin (vitamin B7 and vitamin H) transported by sodium-dependent multivitamin transporters (SMVTs) on BVECs and glioma cells [110,111], and the similar result was obtained as in Glu-Vc-Lip.

##### One ligand + one type of cell membrane camouflage

To overcome the limitations of using single-cell membranes to camouflage nanodrugs, researchers have developed ligand and cell membrane co-modified nanodrug to enhance targeting efficacy and treatment effect on gliomas. For delivering interleukin-12 messenger RNA, Zhao et al.[112] designed a cRGD-decorated GL261 cell membrane (CM) camouflaged calcium carbonate nanodrug delivery system (IL-12 mRNA@cRGD-CM-CaCO_3_ NPs). The cRGD can specifically target the αvβ_3_ integrin, which is highly expressed in GBM neovasculature [113] and some glioma cells [114]. Cellular uptake assay indicated that Cy3-mRNA@cRGD-CM-CaCO_3_ NPs can be more uptaken than Cy3-mRNA@CM-CaCO_3_ NPs because of stronger fluorescence intensity in GL261 cells, suggesting that dual decoration of cRGD and GL261 CM could meaningfully enhance glioma targeting efficacy by the cooperation of the receptor-mediated transporter and the homing/homotypic targeting effect. Furthermore, in vivo imaging track in an intracranial orthotopic glioblastoma (GL261) mice model showed that Luc mRNA@cRGD-CM-CaCO_3_ NPs showed almost 1.6-fold higher bioluminescence signal intensity than Luc mRNA@CM-CaCO_3_ NPs in the glioma area, demonstrating that the cRGD decoration can further improve the BBB crossing and GL261 cell targeting capability of NPs. Finally, the IL-12 mRNA@cRGD-CM-CaCO_3_ NPs exhibited the best orthotopic GBM inhibiting effect and greatly extended the median lifespan of mice with GBM owing to the highest accumulation in tumor area compared with other controls. Similarly, Mo et al. constructed hydroxychloroquine (HDX)-carried yolk-shell upconversion NP (YSN) covering with the cRGD-embedded U87 cell membrane for NIR-triggered treatment toward U87 GBM [115]. Compared with nontargeting and single-targeting decorated nanodrugs, the dual-targeting decorated HDX@YSN@CCM@cRGD showed significantly better U87 cell targeting efficiency in vitro, and better BBB crossing and orthotopic U87 glioblastoma targeting efficiency in vivo. Finally, the HDX@YSN@CCM@cRGD achieved excellent treatment efficacy under the functions of dual targeting, chemotherapy, and PDT in in vitro and in vivo GBM mice models. Recently, Yang et al. [116] utilized nanoplatelets prepared from PM fragments and polyethyleneimine to co-deliver TMZ and miR-375, and modified their surface with RVG-29 peptide, naming it NR/TMZ/miR-375. Due to the dual-targeting ability of both RVG-29 and the nanoplatelets toward the BBB and gliomas, NR/TMZ/miR-375 demonstrated excellent tumor penetration effects in situ glioma therapy, enabling the 2 drugs to exert a synergistic therapeutic effect on the tumor cells and effectively inhibit tumor growth.

##### Two kinds of cell membrane camouflages

**Fig. 4.**
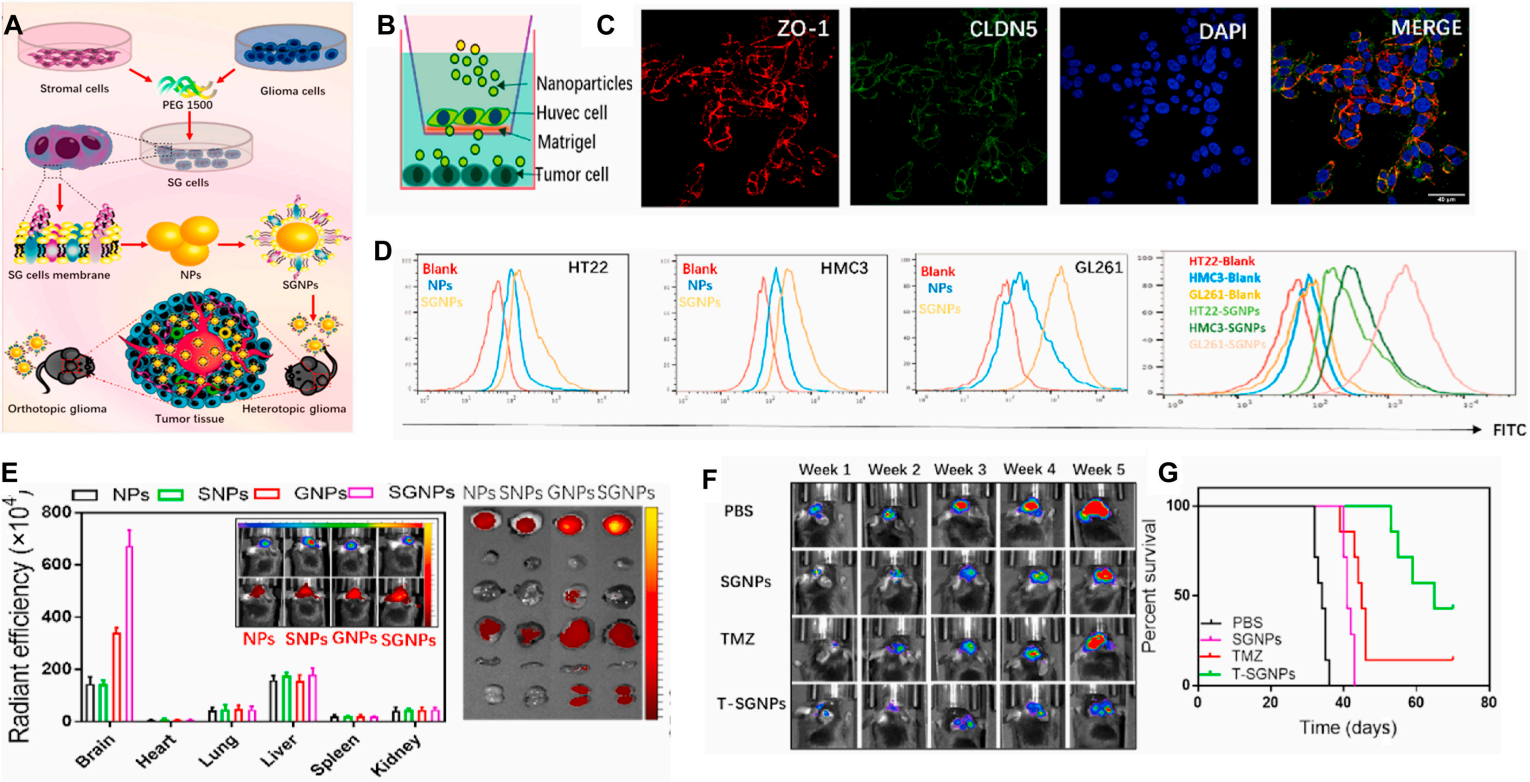
(A) Schematic of preparation of glioma-associated stromal cell (GASC)–glioma cell fusion cell (SG cell) membrane camouflaged PLGA@TMZ nanoparticle (SGNP). (B) Schematic of the TME blood vessels created by the transwell co-culture system. (C) Immunofluorescence of CLDN5 and ZO-1 at the junctional regions of HUVEC monolayers. (D) The uptake difference of SGNPs by HT22, HMC3, and GL-261 cells. (E) Quantification and distribution of IR-780 encapsulated NPs in an orthotopic glioma model. (F) IVIS images of glioma change in the mouse brain under NP treatment. (G) Kaplan–Meier survival curves of mice receiving the different treatments. Reproduced from [117] with permission from the Elsevier, Copyright 2023.

Hybrid cell membranes for endowing complex functions on nanodrugs by fusing membranes from different types of cells are also utilized to improve BBB crossing and glioma targeting ability. Yin et al. [97] built neutrophil and macrophage membranes (NMm) co-coated and RAPA-loaded PLGA nanomedicine (NMm-PLGA/RAPA). An in vitro BBB model demonstrated that more NMm-PLGA/RAPA across the BBB migrated into C6 glioma cells under chemotactic stimulation compared with single membrane-coated NPs. An in vivo glioma inhibition experiment showed that NMm-PLGA/RAPA accumulated in glioma tissues, eliminated glioma cells, and induced durable tumor regression based on RAPA-induced chemotherapy. Ma et al. [117] applied glioma-associated stromal cell (GASC)–glioma cell fusion cell (SG cell) membranes to camouflage PLGA@TMZ NPs to obtain SGNPs. The inherited membrane proteins on the SGNPs markedly improved their BBB crossing efficiency, glioma targeting ability, and glioma cell lethal efficacy in vitro and in vivo in comparison to nanodrugs camouflaged with single-type cell membranes (Fig. 4).

#### One decoration targeting the BBB and another decoration targeting glioma lesions

Because of the expressed diversity of receptors or transporters on 2 different targeted sites (BBB and glioma cells), the united targeting decorations were usually utilized to satisfy the dual-targeting necessary of nanodrugs for the BBB and glioma cells.

##### Two kinds of ligands

As reported, des-octanoyl ghrelin (28 amino acids) transports only from the blood to the brain direction by binding to the ghrelin receptor on BVECs [118,119]. Folate receptors are frequently highly expressed on the cell membrane of C6 glioma cells. Therefore, Chen et al. [120] conjugated des-octanoyl ghrelin and FA to polymersome loading Dox (GFP-D) to satisfy BBB penetrating and glioma targeting, respectively. An in vitro BBB model and an in vivo imaging in mice model with C6 glioma illustrated the obvious dual-targeting effect of GFP-D on the BBB and glioma cells. Compared to other control groups, in vivo anti-C6 glioma studies demonstrated that GFP-D had better tumor development inhibition outcome and exhibited a meaningful extension in the overall lifespan of mice (Fig. S4). Niu et al. [121] constructed glucose/FA co-decorated and Dox-loaded Pluronic P105 polymeric micelles (GF-Dox) for glioma-targeting therapy. The glucose conjugation aimed at targeting GLUT1 and assisting nanodrug crossing the BBB. The FA conjugation was applied to help the nanodrug target glioma cells because of the high expression of FA receptors on the surface of glioma cells. In vivo antitumor studies in C6 glioma-bearing mice showed that the GF-Dox-treated mouse had a minimal tumor volume than other control groups. Similarly, Gao et al. used folate and transferrin to decorate liposomes [122]. Because of the inherent ability of folate and transferrin separately in crossing the BBB and targeting gliomas, the dual-targeting decorated liposome loading Dox finally exhibited excellent antitumor effect by significantly increasing mice survival time and decreasing tumor volume, among others.

### Two decoration-mediated triple-targeting system

Based on the special demand of glioma development, some proteins can be overexpressed on many sites and different types of cell surfaces, such as BVECs (element of the BBB), tumor neovascular endothelial cells (element of the BBTB), and glioma cells, thus providing a greater chance for triple-targeting the BBB, BBTB, and glioma cells. With brain tumor development, the BBB can be damaged and result in the formation of the BBTB, which allows more and bigger molecules to enter brain tissues [10,123]. However, it exhibits highly heterogeneous permeability to nanodrugs [124]. Therefore, targeting and crossing the BBTB is also an important process for nanodrug reaching glioma lesions. Two decoration-mediated triple-targeting functions are becoming popular in nanocarrier-mediated drug delivery in glioma treatment because of their high efficiency. In this case, multiple combinations demonstrate more complex targeted enhancement effects in glioma treatment.

#### Two kinds of ligands

**Fig. 5.**
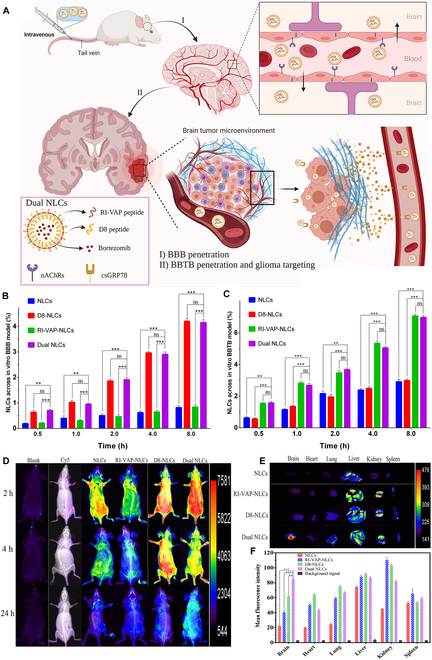
(A) The process of dual NLCs achieving triple-targeting BBB, BBTB, and glioma. Transcytosis efficiency of various NPs loaded with Coumarin 6 in vitro BBB (B) and BBTB (C) models. (D) Whole-body fluorescence imaging of glioma-bearing mice after intravenous injection with Cy5-labeled NPs at 2, 4, and 24 h. (E) Ex vivo fluorescence imaging of glioma-bearing mice, and (F) average fluorescence intensity of the major organs. Reproduced from [53] with permission from Elsevier, Copyright 2022.

Chen et al. [125] established the c(RGDfK) and peptide-22 co-modified liposomal nanodrug [c(RGDfK)/Pep-22-LP] to achieve triple-targeting capabilities for the BBB, BBTB, and glioma. As previously reported, Pep-22 has dual-targeting bioactivity for both BBB and glioma cells by binding to LDLR [64], and c(RGDfK) exhibits dual-targeting capabilities toward the BBTB and glioma cells by binding to integrins α_v_β_3_/α_v_β_5_[113]. In vivo imaging confirmed that c(RGDfK)/Pep-22-LP showed higher accumulation in glioma tissues compared to nanodrug decorated with a single ligand. The median lifespan of glioma-bearing mice treated with Dox-loaded c(RGDfK)/Pep-22-Dox-LP was markedly extended, far exceeding that of mice treated with free Dox or other nanodrugs. Farshbaf et al. [53] constructed D8 peptide and RI-VAP peptide co-decorated nanostructured lipid carriers (Dual NLCs). D8 has a high affinity to nAChRs, which are highly expressed on both BVECs and some glioma cells [126]. RI-VAP as a specific ligand of cell surface GRP78 [127], serving as a selective marker for angiogenesis and cancer cell surfaces, can not only bypass the BBTB but also possess excellent glioma-homing properties [128]. Therefore, dual NLCs have triple-targeting capabilities toward the BBB, BBTB, and glioma. The result from in vitro BBB and BBTB models demonstrated that more dual NCLs can cross the BBB or BBTB than other single-modified or nonmodified NPs. Additionally, in vivo imaging illustrated the strong ability of dual NLCs targeting glioma except for crossing the BBB and BBTB. The dual NLCs@bortezomib exhibited the best glioma cell development inhibition efficiency in vitro and in vivo, and hence, the lifespan of mice with orthotopic glioma was also remarkably extended under the treatment of dual NLCs@bortezomib (Fig. 5).

#### One ligand + one type of cell membrane camouflage

Chen et al. [129] designed an NGR peptide decorated-glioma C6 cancer cell membrane (CCM)-coated dihydroartemisinin nanostructured lipid carrier (DHA-loaded NGR/CCNLC). The NGR peptide has a high affinity to CD13 on endothelial cells and some tumor cells, and the C6 CCM targets C6 glioma cells via a homologous targeting mechanism. In vitro BBB and BBTB models indicated that the NGR/CCNLC had the greatest ability to cross the BBB and BBTB in comparison with CCNLC and nontargeting decorated NLC. In vivo imaging illustrated that more DHA-NGR/CCNLC can enter glioma tissues owing to stronger fluorescence than that in other control groups, which suggests that the biomimetic nanodrug decorated by NGR had excellent BBB/BBTB-crossing and in situ glioma-targeting abilities. Fan et al. [130] developed ^D^WSW peptide-modified and C6 CCM-camouflaged PTX nanosuspensions [^D^WSW-CCM-(PTX)NS]. Under the action of homologous targeting of CCM, the nanodrug gained BBB crossing and tumor targeting function. With the further help of ^D^WSW peptide that can combine with a quorum sensing receptor expressed on endothelial cells [131], the nanodrug was given enhanced BBB/BBTB penetration and tumor-targeting abilities. Cell uptake experiments, an in vitro BBB/BBTB model, and in vivo imaging separately illustrated that ^D^WSW-CCM-(PTX)NS can be more endocytosed by C6 cells, effectively across the BBB/BBTB, and target glioma tissues. Cytotoxicity assays and in vivo glioma therapy demonstrated that ^D^WSW-CCM-(PTX)NS possessed optimal glioma development inhibition effects. Additionally, it prolonged the lifespan of mice with glioma and contributed to triple targeting (BBB, BBTB, and glioma), which induced improved accumulation of nanodrug in glioma cells.

#### Two kinds of cell membrane camouflages

Hao et al. [132] applied C6 CCM and DC membrane (DCm) to create a hybrid membrane and used it to cover DTX nanosuspensions (DNS-[C6&DC]m). The cellular uptake assay showed that DNS-[C6&DC]m was obviously more uptaken by bEnd.3 (member of BBB), HUVECs (member of the BBTB), and C6 glioma cells than single membrane-coated nanosuspensions (DNS-C6m and DNS-DCm). In vitro BBB and BBTB models revealed that the BBB/BBTB crossing abilities of DNS-[C6&DC]m were also markedly stronger than DNS-C6m and DNS-DCm. These results demonstrated that hybrid glioma and DC membrane could achieve triple-targeting efficacy to the BBB, BBTB, and glioma and therefore could further accumulate in glioma lesions and help nanodrug achieve combined chemotherapy and immunotherapy.

## More Targeting Decoration, Better Targeting Efficiency?

As reported in most articles, multi- or dual-targeted modified nanodrugs are usually superior to single-targeted ones. The current comparison methods for dual-targeted vs. single-targeted modified nanomedicines can be mainly divided into 2 forms (Fig. S5).

### Method 1: The cumulative effect of targeting group proportion

Currently, most dual-targeted nanomedicines use a direct additive approach for the proportion of 2 targeting groups. For example, in the study reported in Ref. [108], when preparing the dual-targeted nanodrug Glu-Vc-Lip, the molar ratio of SPC:cholesterol:Glu-Vc-Chol was 62:33:3. Similarly, when preparing the single-targeted nanodrug Glu-Lip or Vc-Lip, the molar ratio of SPC:cholesterol:Glu-Chol or Vc-Chol was also 62:33:3. This indicates that Glu-Vc-Lip contains one more targeting group (Glu or Vc) compared to the single-targeted versions, thus providing more opportunities for interaction with transporters, resulting in higher uptake by cells that highly express GLUT1 (Glu transporter) and sodium-dependent vitamin C transporter 2 (SVCT2, Vc transporter). Similarly, in Ref. [109], the preparation of the dual-targeted nanomedicine Bio_2_ + Glu_3_-Lip used a molar ratio of SPC:cholesterol:Glu_3_-Chol:Bio_2_-Chol of 65:25:5:5, while the single-targeted nanomedicine Bio_2_-Lip or Glu_3_-Lip had molar ratios of SPC:cholesterol:Bio_2_-Chol or Glu_3_-Chol of 65:30:5. This shows that Bio_2_ + Glu_3_-Lip has twice the total amount of targeting groups compared to the single-targeted versions, thus enhancing its uptake by cells with high expression of SMVTs (Bio_2_ transporter) and GLUT1 due to the increased proportion of targeting groups.

However, there is a flaw in this comparison. When a single-targeted approach is not saturated, increasing the dose of the targeting group may achieve similar or even better targeting effects than dual-targeted systems. Therefore, comparing nanomedicines with equal total amounts of targeting groups is more meaningful. For example, the comparison should be made between Bio_2_ + Glu_3_-Lip (SPC:cholesterol:Glu_3_-Chol:Bio_2_-Chol = 65:25:5:5) and Bio_2_-Lip (SPC:cholesterol:Bio_2_-Chol = 65:25:10) or Glu_3_-Lip (SPC:cholesterol:Glu_3_-Chol = 65:25:10). In such cases, dual-targeted modification may not outperform single-targeted modification.

### Method 2: The advantage of targeting diversity

In Ref. [120], dual-targeted modified nanodrug (GFP-D) was studied, where the proportions of 2 targeting groups (folate-PEGGM-PDSGM and des-octanoyl ghrelin-PEGGM-PDSGM) were each half of the corresponding group in single-targeted nanodrugs (FP-D or GP-D). Despite the lower dose of every targeting group, GFP-D accumulated significantly more in brain tumor sites compared to FP-D and GP-D, suggesting that targeting diversity can effectively compensate for the limitations of single-targeted modification. This demonstrates that the advantage of targeting diversity lies in the combination of multiple targeting groups, remarkably improving the targeting ability of nanomedicines and overcoming the limitations of single-targeted modification, such as target saturation and off-target effects.

In particular, when the increase in accumulation of dual-targeted nanomedicines at the tumor site is similar between 2 approaches compared to single-targeted nanomedicines, the second approach with dual targets is more advantageous and has higher application value. If, in the first approach, the accumulation of dual-targeted nanomedicines at the tumor site is only twice that of the single-targeted version, this effect might result solely from the dose accumulation of targeting groups, meaning that dual-targeted modification does not offer a significant advantage over single-targeted modification, and thus, its application value is limited. If a second approach employs triple or more targeting modifications, the diversity of targets can overcome saturation and off-target effects, further enhancing targeting accuracy and significantly increasing the accumulation of nanomedicines in the tumor site, thereby improving therapeutic efficacy.

In conclusion, whether more targeting decorated nanomedicines are superior depends on the synergistic effects of cumulative targeting group doses and targeting diversity. When the targeting group total doses are equal, the second approach’s advantage of targeting diversity becomes more evident, while the first approach’s effect is more reliant on the cumulative doses of targeting groups, although the idea of increasing the type or number of targeting modifications to enhance targeting efficacy remains viable. However, as the number of modifications increases, the preparation process becomes more complicated, and the technical requirements may not be met. Additionally, an excessive number of external modifications may pose safety concerns for the organism.

## Conclusion and Future Perspectives

In this review, we provide a detailed explanation of the combined strategies for noninvasively overcoming the BBB and actively targeting glioma lesions using nanodrug delivery systems. Whether through a reversible temporary opening of the BBB or a nose-to-brain route that bypasses the BBB, once nanodrugs enter the brain parenchyma, tumor-specific targeting modifications on their surface are required to ensure rapid and precise accumulation in glioma lesions. These modifications can consist of whole cells, ligands, or cell membrane fragments. If the strategy involves crossing endothelial cells to overcome the BBB, the surface of the nanodrugs must also carry targeting modifications specific to BBB endothelial cells, which can similarly be whole cells, ligands, or cell membrane fragments. If a single surface modification can both facilitate nanodrug passage across BBB endothelial cells and recognize glioma lesions, this is referred to as a “single decoration-mediated dual-targeting system”. Conversely, if 2 different surface modifications are needed to achieve BBB penetration and glioma lesion recognition, this is called a “2 decoration-mediated dual-targeting system”. Compared to the “2 decoration-mediated dual-targeting system”, the “single decoration-mediated dual-targeting system” can achieve dual objectives with simpler modifications. However, this does not necessarily guarantee optimal targeting performance. Due to differences in receptor or transporter expression between endothelial cells and glioma cells [133], a single targeting modification often struggles to achieve the best targeting effect at both sites. In contrast, dual-targeting modifications allow each modification to optimize targeting at its respective site. Although the preparation of this combined approach is slightly more complex, its targeting efficiency is relatively superior [115].

In the “2 decorations simultaneously targeting both BBB and glioma lesions” system, each targeting modification can simultaneously target 2 sites, thus collectively mediating quadruple targeting. In contrast, in the “one decoration targeting the BBB and another decoration targeting glioma lesions” system, each targeting modification can only target one site, resulting in dual targeting from the 2 modifications. Theoretically, this gives the former an advantage over the latter in the selection of nanodrugs for glioma treatment, as the increased targeting relationships can enhance the accumulation of nanodrugs in glioma lesions. However, there is currently a lack of direct studies comparing these 2 systems. Furthermore, compared to the “2 decoration-mediated dual-targeting system”, the “2 decoration-mediated triple-targeting system” can help nanodrugs overcome more barriers, such as the BBTB, while also potentially exhibiting dual or even triple targeting effects from each modification [130]. This leads to a complex multitargeting system that can further increase the probability of nanodrugs breaking through barriers to reach glioma lesions.

Even though the combination of noninvasive BBB overcoming and active glioma lesion targeting strategies has achieved good coordination and obtained excellent results in nanodrug-mediated glioma treatment, there is still room for improvement. The following aspects can be considered to improve nanodrug delivery systems, enhance safety, and drive clinical translation of nanodrug in glioma treatment: (a) Compared to passively passing BBB, nanodrug actively and selectively crossing the BBB using targeted decoration can further enhance accuracy and safety. However, because of various obstructions of nanodrug in blood circulation, there are higher requirements for the material of nanocarriers and specificity of targeting decoration groups in nanodrug delivery systems. For example, surface positively charged nanocarriers are easy to accumulate in the kidney to produce toxic side effects, while biologically derived nanocarriers have lower side effects [134]. In addition to being highly expressed in glioma tissues and the BBB, some receptors or transporters also slightly overexpressed in some organs or tissues to satisfy physiological needs, which hence declines the targeted specificity. Therefore, further understanding the distribution of targets in vivo and discovering new targets with high specificity are continuing challenges in the area of targeted drug delivery. (b) The BBTB plays a vital role in glioma development and is also a key challenge (barrier) that needs to be overcome using nanodrug owing to its difference from BBB and heterogeneous permeability to nanodrug [22,135]. However, the BBTB is usually ignored in many studies. Importantly, new or key targeted sites on the BBTB need to be further explored so that there is more chance for nanodrug to effectively cross the BBTB. Furthermore, if the same target site on the BBB, BBTB, and glioma lesions can be applied for a single decorated nanodrug to achieve “one decoration-mediated triple targeting”, not only the targeted decoration of nanodrug can be simplified, but also the targeted efficacy of nanodrug may also be improved, contributing to focused targeting of the “3 birds, 1 stone” strategy. (c) The combination of immunotherapy with nanodrug is a promising strategy in glioma therapy because of the activation of immune cells and their inherent ability to attack glioma cells [136]. Whether using activated immune cells to deliver nanodrugs or using ICMs to camouflage nanodrugs, cooperating drug and immune therapy can exert synergistic effects to further enhance the glioma-killing effect. In the future, new and more effective combinations of immunotherapy and drugs are worth exploring.

Although nanodrugs have undergone several decades of preclinical research for the treatment of gliomas, mainly lipid-based nanodrugs have currently entered clinical trials. Clinical studies show that while some lipid nanodrugs, particularly PEGylated ones, can prolong progression-free intervals of glioma, they cannot extend the survival of glioma patients and do not provide a curative effect [137]. In 2014, the clinical Phase I/II trials were completed using glutathione-decorated PEGylated liposomal Dox, named 2B3-101 [138]. Previous studies have shown that glutathione can help nanodrugs cross the BBB by targeting the glutathione transporter [139]. The clinical result exhibited that compared to PEGylated liposomal Dox without glutathione decoration, 2B3-101 significantly enhanced the accumulation of Dox in recurrent malignant gliomas. However, targeting modified nanodrugs for active targeting of the BBB and glioma for glioma treatment are still rare in preclinical research. The slow progress of nanodrugs in clinical therapy for gliomas can be attributed to several main reasons: (a) In preclinical studies, the research on the side effects of nanomaterials is insufficient, often relying solely on simple characterization to infer their biocompatibility. Nanotoxicology is an area that requires deeper exploration, and researchers should focus more on the biocompatibility of nanodrugs rather than merely pursuing scientific innovation. (b) The target specificity of active targeting is insufficient, which may lead to off-target effects, and the associated side effects are still unclear. (c) Despite various targeted modifications, the percentage of nanodrugs that can effectively reach glioma lesions is quite low. (d) Many nanodrugs lack mature large-scale production technologies, resulting in higher costs.

In summary, the clinical translation of nanodrugs in the treatment of solid gliomas in situ still has a long way to go, but continuous optimization and improvement from nanocarriers, targeted modifications, and treatment modalities give the field constant hope.

## Data Availability

This review has no data that need to be uploaded.

## Supplementary Materials

Supplementary 1 Figs. S1 to S5 Tables S1 and S2 References [140,141]

## References

[1] Barnholtz-Sloan JS , Ostrom QT , Cote D . Epidemiology of brain tumors. Neurol Clin. 2018;36(3):395–419. 30072062 10.1016/j.ncl.2018.04.001

[2] Louis DN , Ohgaki H , Wiestler OD , Cavenee WK , Burger PC , Jouvet A , Scheithauer BW , Kleihues P . The 2007 WHO classification of tumours of the central nervous system. Acta Neuropathol. 2007;114:97–109. 17618441 10.1007/s00401-007-0243-4 PMC1929165

[3] El Atat O , Naser R , Abdelkhalek M , Habib RA , El Sibai M . Molecular targeted therapy: A new avenue in glioblastoma treatment. Oncol Lett. 2023;25(2):1–33. 36644133 10.3892/ol.2022.13632 PMC9811647

[4] Aldape K , Zadeh G , Mansouri S , Reifenberger G , von Deimling A . Glioblastoma: Pathology, molecular mechanisms and markers. Acta Neuropathol. 2015;129(6):829–848. 25943888 10.1007/s00401-015-1432-1

[5] Wen PY , Kesari S . Malignant gliomas in adults. New Engl J Med. 2008;359(5):492–507. 18669428 10.1056/NEJMra0708126

[6] Hanif F , Muzaffar K , Perveen K , Malhi SM , Simjee SU . Glioblastoma multiforme: A review of its epidemiology and pathogenesis through clinical presentation and treatment. Asian Pac J Cancer Prev. 2017;18(1):3–9. 28239999 10.22034/APJCP.2017.18.1.3 PMC5563115

[7] Toader C , Eva L , Costea D , Corlatescu AD , Covache-Busuioc RA , Bratu BG , Glavan LA , Costin HP , Popa AA , Ciurea AV . Low-grade gliomas: Histological subtypes, molecular mechanisms, and treatment strategies. Brain Sci. 2023;13(12):1700. 38137148 10.3390/brainsci13121700 PMC10741942

[8] McKinnon C , Nandhabalan M , Murray SA , Plaha P . Glioblastoma: Clinical presentation, diagnosis, and management. BMJ. 2021;374:Article n1560. 34261630 10.1136/bmj.n1560

[9] Raucher D , Dragojevic S , Ryu J . Macromolecular drug carriers for targeted glioblastoma therapy: Preclinical studies, challenges, and future perspectives. Front Oncol. 2018;8:624. 30619758 10.3389/fonc.2018.00624 PMC6304427

[10] Bellotti E , Schilling AL , Little SR , Decuzzi P . Injectable thermoresponsive hydrogels as drug delivery system for the treatment of central nervous system disorders: A review. J Control Release. 2021;329:16–35. 33259851 10.1016/j.jconrel.2020.11.049

[11] Pardridge WM . Blood-brain barrier delivery. Drug Discov Today. 2007;12(1–2):54–61. 17198973 10.1016/j.drudis.2006.10.013

[12] Li J , Zhao J , Tan T , Liu M , Zeng Z , Zeng Y , Zhang L , Fu C , Chen D , Xie T . Nanoparticle drug delivery system for glioma and its efficacy improvement strategies: A comprehensive review. Int J Nanomedicine. 2020;15:2563–2582. 32368041 10.2147/IJN.S243223 PMC7173867

[13] Akhter MH , Rizwanullah M , Ahmad J , Amin S , Ahmad MZ , Minhaj MA , Mujtaba MA , Ali J . Molecular targets and nanoparticulate systems designed for the improved therapeutic intervention in glioblastoma multiforme. Drug Res. 2021;71(3):122–137. 10.1055/a-1296-7870 33167048

[14] Marchetti L , Engelhardt B . Immune cell trafficking across the blood-brain barrier in the absence and presence of neuroinflammation. Vasc Biol. 2020;2(1):H1–H18. 32923970 10.1530/VB-19-0033 PMC7439848

[15] Wu SK , Tsai CL , Huang Y , Hynynen K . Focused ultrasound and microbubbles-mediated drug delivery to brain tumor. Pharmaceutics. 2020;13(1):15. 33374205 10.3390/pharmaceutics13010015 PMC7823947

[16] Bruinsmann FA , Richter Vaz G , de Cristo Soares Alves A , Aguirre T , Raffin Pohlmann A , Stanisçuaski Guterres S , Sonvico F . Nasal drug delivery of anticancer drugs for the treatment of glioblastoma: Preclinical and clinical trials. Molecules. 2019;24(23):4312. 31779126 10.3390/molecules24234312 PMC6930669

[17] Van Woensel M , Wauthoz N , Rosière R , Amighi K , Mathieu V , Lefranc F , Van Gool SW , De Vleeschouwer S . Formulations for intranasal delivery of pharmacological agents to combat brain disease: A new opportunity to tackle GBM? Cancers. 2013;5(3):1020–1048. 24202332 10.3390/cancers5031020 PMC3795377

[18] Sandbhor P , Goda J , Mohanty B , Gera P , Yadav S , Chekuri G , Chaudhari P , Dutt S , Banerjee R . Targeted nano-delivery of chemotherapy via intranasal route suppresses in vivo glioblastoma growth and prolongs survival in the intracranial mouse model. Drug Delivery Transl Res. 2023;13(2):608–626. 10.1007/s13346-022-01220-8 36245060

[19] Chu L , Wang A , Ni L , Yan X , Song Y , Zhao M , Sun K , Mu H , Liu S , Wu Z , et al. Nose-to-brain delivery of temozolomide-loaded PLGA nanoparticles functionalized with anti-EPHA3 for glioblastoma targeting. Drug Deliv. 2018;25(1):1634–1641. 30176744 10.1080/10717544.2018.1494226 PMC6127843

[20] Kanazawa T , Taki H , Okada H . Nose-to-brain drug delivery system with ligand/cell-penetrating peptide-modified polymeric nano-micelles for intracerebral gliomas. Eur J Pharm Biopharm. 2020;152:85–94. 32387702 10.1016/j.ejpb.2020.05.001

[21] Nwafor DC , Obiri-Yeboah D , Fazad F , Blanks W , Mut M . Focused ultrasound as a treatment modality for gliomas. Front Neurol. 2024;15:1387986. 38813245 10.3389/fneur.2024.1387986 PMC11135048

[22] Marcucci F , Corti A , Ferreri AJM . Breaching the blood-brain tumor barrier for tumor therapy. Cancers. 2021;13(10):2391. 34063335 10.3390/cancers13102391 PMC8156088

[23] Ho Y-J , Huang C-C , Fan C-H , Liu H-L , Yeh C-K . Ultrasonic technologies in imaging and drug delivery. Cell Mol Life Sci. 2021;78:6119–6141. 34297166 10.1007/s00018-021-03904-9 PMC11072106

[24] Fateh ST , Moradi L , Kohan E , Hamblin MR , Dezfuli AS . Comprehensive review on ultrasound-responsive theranostic nanomaterials: Mechanisms, structures and medical applications. Beilstein J Nanotechnol. 2021;12(1):808–862. 34476167 10.3762/bjnano.12.64 PMC8372309

[25] Fishman PS , Frenkel V . Focused ultrasound: An emerging therapeutic modality for neurologic disease. Neurotherapeutics. 2017;14:393–404. 28244011 10.1007/s13311-017-0515-1 PMC5398988

[26] Cai Q , Li X , Xiong H , Fan H , Gao X , Vemireddy V , Margolis R , Li J , Ge X , Giannotta M , et al. Optical blood-brain-tumor barrier modulation expands therapeutic options for glioblastoma treatment. Nat Commun. 2023;14(1):4934. 37582846 10.1038/s41467-023-40579-1 PMC10427669

[27] Zhang X , Ye D , Yang L , Yue Y , Sultan D , Pacia CP , Pang H , Detering L , Heo GS , Luehmann H , et al. Magnetic resonance imaging-guided focused ultrasound-based delivery of radiolabeled copper nanoclusters to diffuse intrinsic pontine glioma. ACS Appl Nano Mater. 2020;3(11):11129–11134. 34337344 10.1021/acsanm.0c02297 PMC8320805

[28] Wu JR , Hernandez Y , Miyasaki KF , Kwon EJ . Engineered nanomaterials that exploit blood-brain barrier dysfunction for delivery to the brain. Adv Drug Del Rev. 2023;197: Article 114820. 10.1016/j.addr.2023.114820 PMC12834260 37054953

[29] McMahon D , Hynynen K . Acute inflammatory response following increased blood-brain barrier permeability induced by focused ultrasound is dependent on microbubble dose. Theranostics. 2017;7(16):3989–4000. 29109793 10.7150/thno.21630 PMC5667420

[30] Zhao G , Huang Q , Wang F , Zhang X , Hu J , Tan Y , Huang N , Wang Z , Wang Z , Cheng Y . Targeted shRNA-loaded liposome complex combined with focused ultrasound for blood brain barrier disruption and suppressing glioma growth. Cancer Lett. 2018;418:147–158. 29339208 10.1016/j.canlet.2018.01.035

[31] Yang Q , Zhou Y , Chen J , Huang N , Wang Z , Cheng Y . Gene therapy for drug-resistant glioblastoma via lipid-polymer hybrid nanoparticles combined with focused ultrasound. Int J Nanomedicine. 2021;16:185–199. 33447034 10.2147/IJN.S286221 PMC7802796

[32] Yang F-Y , Wong T-T , Teng M-C , Liu R-S , Lu M , Liang H-F , Wei M-C . Focused ultrasound and interleukin-4 receptor-targeted liposomal doxorubicin for enhanced targeted drug delivery and antitumor effect in glioblastoma multiforme. J Control Release. 2012;160(3):652–658. 22405901 10.1016/j.jconrel.2012.02.023

[33] Habib S , Singh M . Angiopep-2-modified nanoparticles for brain-directed delivery of therapeutics: A review. Polymers. 2022;14(4):712. 35215625 10.3390/polym14040712 PMC8878382

[34] Wang S , Meng Y , Li C , Qian M , Huang R . Receptor-mediated drug delivery systems targeting to glioma. Nanomaterials. 2015;6(1):3. 28344260 10.3390/nano6010003 PMC5302535

[35] Hosseinalizadeh H , Mahmoodpour M , Bahabadi ZR , Hamblin MR , Mirzaei H . Neutrophil mediated drug delivery for targeted glioblastoma therapy: A comprehensive review. Biomed Pharmacother. 2022;156:Article 113841. 36411657 10.1016/j.biopha.2022.113841

[36] Xue J , Zhao Z , Zhang L , Xue L , Shen S , Wen Y , Wei Z , Wang L , Kong L , Sun H , et al. Neutrophil-mediated anticancer drug delivery for suppression of postoperative malignant glioma recurrence. Nat Nanotechnol. 2017;12(7):692–700. 28650441 10.1038/nnano.2017.54

[37] Yang L , Zhang K , Zheng D , Bai Y , Yue D , Wu L , Ling H , Ni S , Zou H , Ye B , et al. Platelet-based nanoparticles with stimuli-responsive for anti-tumor therapy. Int J Nanomedicine. 2023;18:6293–6309. 37954456 10.2147/IJN.S436373 PMC10637234

[38] Lin YJ , Wei KC , Chen PY , Lim M , Hwang TL . Roles of neutrophils in glioma and brain metastases. Front Immunol. 2021;12:Article 701383. 34484197 10.3389/fimmu.2021.701383 PMC8411705

[39] Ding M , Zhu A , Zhang Y , Liu J , Lin L , Wang X , Li J . Neutrophil-based Trojan horse containing polymer nano-therapeutics for sono-activatable ferroptosis-immunotherapy of orthotopic glioma. Nano Today. 2024;57:Article 102398.

[40] Peng Y , Chen F , Li S , Liu X , Wang C , Yu C , Li W . Tumor-associated macrophages as treatment targets in glioma. Brain Sci Adv. 2021;6:306–323.

[41] Wang S , Shen H , Mao Q , Tao Q , Yuan G , Zeng L , Chen Z , Zhang Y , Cheng L , Zhang J , et al. Macrophage-mediated porous magnetic nanoparticles for multimodal imaging and postoperative photothermal therapy of gliomas. ACS Appl Mater Interfaces. 2021;13(48):56825–56837. 34825820 10.1021/acsami.1c12406

[42] Ibarra LE , Beaugé L , Arias-Ramos N , Rivarola VA , Chesta CA , López-Larrubia P , Palacios RE . Trojan horse monocyte-mediated delivery of conjugated polymer nanoparticles for improved photodynamic therapy of glioblastoma. Nanomedicine. 2020;15(17):1687–1707. 32689873 10.2217/nnm-2020-0106

[43] Zhou J , Li L , Jia M , Liao Q , Peng G , Luo G , Zhou Y . Dendritic cell vaccines improve the glioma microenvironment: Influence, challenges, and future directions. Cancer Med. 2023;12(6):7207–7221. 36464889 10.1002/cam4.5511 PMC10067114

[44] Li T-F , Li K , Zhang Q , Wang C , Yue Y , Chen Z , Yuan S-J , Liu X , Wen Y , Han M , et al. Dendritic cell-mediated delivery of doxorubicin-polyglycerol-nanodiamond composites elicits enhanced anti-cancer immune response in glioblastoma. Biomaterials. 2018;181:35–52. 30071380 10.1016/j.biomaterials.2018.07.035

[45] Jiang Z , Zhang H , Zhang W , Zhang Y , Cui Y , Mei L , Wang Q . Smart platelet-based biohybrid delivery system for magnetic-guided targeted delivery and enhanced photothermal-chemo therapy against glioma. Nano Today. 2024;56:Article 102295.

[46] Zhang D , Tian S , Liu Y , Zheng M , Yang X , Zou Y , Shi B , Luo L . Near infrared-activatable biomimetic nanogels enabling deep tumor drug penetration inhibit orthotopic glioblastoma. Nat Commun. 2022;13(1):6835. 36369424 10.1038/s41467-022-34462-8 PMC9652403

[47] Su J , Yao Z , Chen Z , Zhou S , Wang Z , Xia H , Liu S , Wu Y . TfR aptamer enhanced blood-brain barrier penetration of biomimetic nanocomplexes for intracellular transglutaminase 2 imaging and silencing in glioma. Small. 2022;18(40):2203448. 10.1002/smll.202203448 35980938

[48] Kusmierz CD , Callmann CE , Kudruk S , Distler ME , Mirkin CA . Transferrin aptamers increase the in vivo blood-brain barrier targeting of protein spherical nucleic acids. Bioconjug Chem. 2022;33(10):1803–1810. 36194889 10.1021/acs.bioconjchem.2c00389 PMC10424462

[49] Janjua TI , Rewatkar P , Ahmed-Cox A , Saeed I , Mansfeld FM , Kulshreshtha R , Kumeria T , Ziegler DS , Kavallaris M , Mazzieri R , et al. Frontiers in the treatment of glioblastoma: Past, present and emerging. Adv Drug Del Rev. 2021;171: 108–138. 10.1016/j.addr.2021.01.012 33486006

[50] McCord E , Pawar S , Koneru T , Tatiparti K , Sau S , Iyer AK . Folate receptors’ expression in gliomas may possess potential nanoparticle-based drug delivery opportunities. ACS Omega. 2021;6(6):4111–4118. 33623837 10.1021/acsomega.0c05500 PMC7893640

[51] Anthony DP , Hegde M , Shetty SS , Rafic T , Mutalik S , Rao BS . Targeting receptor-ligand chemistry for drug delivery across blood-brain barrier in brain diseases. Life Sci. 2021;274:Article 119326. 33711385 10.1016/j.lfs.2021.119326

[52] Li J , Du Y , Jiang Z , Tian Y , Qiu N , Wang Y , Hu M , Zou R , Luo L , Du S . Y1 receptor ligand-based nanomicelle as a novel nanoprobe for glioma-targeted imaging and therapy. Nanoscale. 2018;10(13):5845–5851. 29542782 10.1039/c8nr00148k

[53] Farshbaf M , Mojarad-Jabali S , Hemmati S , Khosroushahi AY , Motasadizadeh H , Zarebkohan A , Valizadeh H . Enhanced BBB and BBTB penetration and improved anti-glioma behavior of bortezomib through dual-targeting nanostructured lipid carriers. J Control Release. 2022;345:371–384. 35301054 10.1016/j.jconrel.2022.03.019

[54] Shi W , Cui X , Shi J , Chen J , Wang Y . Overcoming the blood-brain barrier for glioma-targeted therapy based on an interleukin-6 receptor-mediated micelle system. RSC Adv. 2017;7(44):27162–27169.

[55] Yeh W-L , Lin C-J , Fu W-M . Enhancement of glucose transporter expression of brain endothelial cells by vascular endothelial growth factor derived from glioma exposed to hypoxia. Mol Pharmacol. 2008;73(1):170–177. 17942749 10.1124/mol.107.038851

[56] Roberts L , Black D , Raman C , Woodford K , Zhou M , Haggerty J , Yan A , Cwirla S , Grindstaff K . Subcellular localization of transporters along the rat blood-brain barrier and blood-cerebral-spinal fluid barrier by in vivo biotinylation. Neuroscience. 2008;155(2):423–438. 18619525 10.1016/j.neuroscience.2008.06.015

[57] Zhang Y , Cheng Q , Xue Y , Yao K , Syeda MZ , Xu J , Wu J , Wang Z , Tang L , Mu Q . LAT1 targeted brain delivery of temozolomide and sorafenib for effective glioma therapy. Nano Res. 2023;16:9743–9751.

[58] Wu X , Prasad PD , Leibach FH , Ganapathy V . cDNA sequence, transport function, and genomic organization of human OCTN2, a new member of the organic cation transporter family. Biochem Biophys Res Commun. 1998;246(3):589–595. 9618255 10.1006/bbrc.1998.8669

[59] Kou L , Hou Y , Yao Q , Guo W , Wang G , Wang M , Fu Q , He Z , Ganapathy V , Sun J . L-carnitine-conjugated nanoparticles to promote permeation across blood-brain barrier and to target glioma cells for drug delivery via the novel organic cation/carnitine transporter OCTN2. Artif Cells, Nanomed Biotechnol. 2018;46(8):1605–1616. 28974108 10.1080/21691401.2017.1384385

[60] Vieira DB , Gamarra LF . Getting into the brain: Liposome-based strategies for effective drug delivery across the blood-brain barrier. Int J Nanomedicine. 2016;11:5381–5414. 27799765 10.2147/IJN.S117210 PMC5077137

[61] Delač M , Motaln H , Ulrich H , Lah TT . Aptamer for imaging and therapeutic targeting of brain tumor glioblastoma. Cytometry A. 2015;87(9):806–816. 26189784 10.1002/cyto.a.22715

[62] Ramalho MJ , Loureiro JA , Coelho MA , Pereira MC . Transferrin receptor-targeted nanocarriers: Overcoming barriers to treat glioblastoma. Pharmaceutics. 2022;14(2):279. 35214012 10.3390/pharmaceutics14020279 PMC8880499

[63] Xie J , Shen Z , Anraku Y , Kataoka K , Chen X . Nanomaterial-based blood-brain-barrier (BBB) crossing strategies. Biomaterials. 2019;224:Article 119491. 31546096 10.1016/j.biomaterials.2019.119491 PMC6915305

[64] Zhang B , Sun X , Mei H , Wang Y , Liao Z , Chen J , Zhang Q , Hu Y , Pang Z , Jiang X . LDLR-mediated peptide-22-conjugated nanoparticles for dual-targeting therapy of brain glioma. Biomaterials. 2013;34(36):9171–9182. 24008043 10.1016/j.biomaterials.2013.08.039

[65] Ye Z , Zhang T , He W , Jin H , Liu C , Yang Z , Ren J . Methotrexate-loaded extracellular vesicles functionalized with therapeutic and targeted peptides for the treatment of glioblastoma multiforme. ACS Appl Mater Interfaces. 2018;10(15):12341–12350. 29564886 10.1021/acsami.7b18135

[66] Pizzocri M , Re F , Stanzani E , Formicola B , Tamborini M , Lauranzano E , Ungaro F , Rodighiero S , Francolini M , Gregori M , et al. Radiation and adjuvant drug-loaded liposomes target glioblastoma stem cells and trigger in-situ immune response. Neurooncol Adv. 2021;3(1):vdab076. 34377986 10.1093/noajnl/vdab076 PMC8349181

[67] Najafi F , Moghimi HR , Hemmati M , Deevband MR , Kazemi B . SRL-coated PAMAM dendrimer nano-carrier for targeted gene delivery to the glioma cells and competitive inhibition by lactoferrin. Iran J Pharm Res. 2016;15(4):629–640. 28243262 PMC5316243

[68] Ruan H , Chai Z , Shen Q , Chen X , Su B , Xie C , Zhan C , Yao S , Wang H , Zhang M , et al. A novel peptide ligand RAP12 of LRP1 for glioma targeted drug delivery. J Control Release. 2018;279:306–315. 29679668 10.1016/j.jconrel.2018.04.035

[69] Ruan H , Yao S , Wang S , Wang R , Xie C , Guo H , Lu W . Stapled RAP12 peptide ligand of LRP1 for micelles-based multifunctional glioma-targeted drug delivery. Chem Eng J. 2021;403:Article 126296.

[70] He W , Li X , Morsch M , Ismail M , Liu Y , Rehman FU , Zhang D , Wang Y , Zheng M , Chung R , et al. Brain-targeted codelivery of Bcl-2/Bcl-xl and Mcl-1 inhibitors by biomimetic nanoparticles for orthotopic glioblastoma therapy. ACS Nano. 2022;16(4):6293–6308. 35353498 10.1021/acsnano.2c00320

[71] Zheng M , Liu Y , Wang Y , Zhang D , Zou Y , Ruan W , Yin J , Tao W , Park JB , Shi B . ROS-responsive polymeric siRNA nanomedicine stabilized by triple interactions for the robust glioblastoma combinational RNAi therapy. Adv Mater. 2019;31(37):1903277. 10.1002/adma.201903277 31348581

[72] Liu Y , Zheng M , Jiao M , Yan C , Xu S , Du Q , Morsch M , Yin J , Shi B . Polymeric nanoparticle mediated inhibition of miR-21 with enhanced miR-124 expression for combinatorial glioblastoma therapy. Biomaterials. 2021;276:Article 121036. 34329919 10.1016/j.biomaterials.2021.121036

[73] Jiang Y , Zhang J , Meng F , Zhong Z . Apolipoprotein E peptide-directed chimeric polymersomes mediate an ultrahigh-efficiency targeted protein therapy for glioblastoma. ACS Nano. 2018;12(11):11070–11079. 30395440 10.1021/acsnano.8b05265

[74] Sun P , Xiao Y , Di Q , Ma W , Ma X , Wang Q , Chen W . Transferrin receptor-targeted PEG-PLA polymeric micelles for chemotherapy against glioblastoma multiforme. Int J Nanomedecine. 2020;15:6673–6688. 10.2147/IJN.S257459 PMC7494234 32982226

[75] Liu X , Cao Z , Liu N , Gao G , Du M , Wang Y , Cheng B , Zhu M , Jia B , Pan L . Kill two birds with one stone: Engineered exosome-mediated delivery of cholesterol modified YY1-siRNA enhances chemoradiotherapy sensitivity of glioblastoma. Front Pharmacol. 2022;13:Article 975291. 36059990 10.3389/fphar.2022.975291 PMC9438942

[76] Yue J , Liu S , Wang R , Hu X , Xie Z , Huang Y , Jing X . Fluorescence-labeled immunomicelles: Preparation, in vivo biodistribution, and ability to cross the blood-brain barrier. Macromol Biosci. 2012;12(9):1209–1219. 22807211 10.1002/mabi.201200037

[77] Liu G , Shen H , Mao J , Zhang L , Jiang Z , Sun T , Lan Q , Zhang Z . Transferrin modified graphene oxide for glioma-targeted drug delivery: In vitro and in vivo evaluations. ACS Appl Mater Interfaces. 2013;5(15):6909–6914. 23883622 10.1021/am402128s

[78] Guo J , Schlich M , Cryan JF , O’Driscoll CM . Targeted drug delivery via folate receptors for the treatment of brain cancer: Can the promise deliver? J Pharm Sci. 2017;106(12):3413–3420. 28842300 10.1016/j.xphs.2017.08.009

[79] Afzalipour R , Khoei S , Khoee S , Shirvalilou S , Jamali Raoufi N , Motevalian M , Karimi MR . Dual-targeting temozolomide loaded in folate-conjugated magnetic triblock copolymer nanoparticles to improve the therapeutic efficiency of rat brain gliomas. ACS Biomater Sci Eng. 2019;5(11):6000–6011. 33405722 10.1021/acsbiomaterials.9b00856

[80] Wang Y , Su Y , Yang Y , Jin H , Wu M , Wang Q , Sun P , Zhang J , Yang X , Shu X . Increased brain uptake of pterostilbene loaded folate modified micellar delivery system. Drug Deliv. 2022;29(1):3071–3086. 36131589 10.1080/10717544.2022.2126559 PMC9848421

[81] Wei X , Zhan C , Shen Q , Fu W , Xie C , Gao J , Peng C , Zheng P , Lu W . A D-peptide ligand of nicotine acetylcholine receptors for brain-targeted drug delivery. Angew Chem. 2015;127(10):3066–3070. 10.1002/anie.201411226 25600241

[82] Zhang D , Kong J , Huang X , Zeng J , Du Q , Yang T , Yue H , Bao Q , Miao Y , Xu Y . Targeted glioblastoma therapy by integrating brain-targeting peptides and corn-derived cancer cell-penetrating proteins into nanoparticles to cross blood-brain tumor barriers. Mater Today Nano. 2023;23:Article 100347.

[83] Jiang X , Xin H , Ren Q , Gu J , Zhu L , Du F , Feng C , Xie Y , Sha X , Fang X . Nanoparticles of 2-deoxy-D-glucose functionalized poly(ethylene glycol)-co-poly(trimethylene carbonate) for dual-targeted drug delivery in glioma treatment. Biomaterials. 2014;35(1):518–529. 24125772 10.1016/j.biomaterials.2013.09.094

[84] Wang S , An J , Dong W , Wang X , Sheng J , Jia Y , He Y , Ma X , Wang J , Yu D , et al. Glucose-coated berberine nanodrug for glioma therapy through mitochondrial pathway. Int J Nanomed. 2020;15:7951–7965. 10.2147/IJN.S213079 PMC7569050 33116511

[85] Li L , Di X , Zhang S , Kan Q , Liu H , Lu T , Wang Y , Fu Q , Sun J , He Z . Large amino acid transporter 1 mediated glutamate modified docetaxel-loaded liposomes for glioma targeting. Colloids Surf B Biointerfaces. 2016;141:260–267. 26859117 10.1016/j.colsurfb.2016.01.041

[86] Fang RH , Kroll AV , Gao W , Zhang L . Cell membrane coating nanotechnology. Adv Mater. 2018;30(23):1706759. 10.1002/adma.201706759 PMC5984176 29582476

[87] Li R , He Y , Zhang S , Qin J , Wang J . Cell membrane-based nanoparticles: A new biomimetic platform for tumor diagnosis and treatment. Acta Pharm Sin B. 2018;8(1):14–22. 29872619 10.1016/j.apsb.2017.11.009 PMC5985624

[88] Li H , Li S , Lin Y , Chen S , Yang L , Huang X , Wang H , Yu X , Zhang L . Artificial exosomes mediated spatiotemporal-resolved and targeted delivery of epigenetic inhibitors. J Nanobiotechnol. 2021;19(1):364. 10.1186/s12951-021-01107-9 PMC8597284 34789273

[89] Wan X , Song L , Pan W , Zhong H , Li N , Tang B . Tumor-targeted cascade nanoreactor based on metal-organic frameworks for synergistic ferroptosis-starvation anticancer therapy. ACS Nano. 2020;14(9):11017–11028. 32786253 10.1021/acsnano.9b07789

[90] Zhao Y-Z , Shen B-X , Li X-Z , Tong M-Q , Xue P-P , Chen R , Yao Q , Chen B , Xiao J , Xu H-L . Tumor cellular membrane camouflaged liposomes as a non-invasive vehicle for genes: Specific targeting toward homologous gliomas and traversing the blood-brain barrier. Nanoscale. 2020;12(28):15473–15494. 32667375 10.1039/d0nr04212a

[91] Chen Z , Zhao P , Luo Z , Zheng M , Tian H , Gong P , Gao G , Pan H , Liu L , Ma A , et al. Cancer cell membrane-biomimetic nanoparticles for homologous-targeting dual-modal imaging and photothermal therapy. ACS Nano. 2016;10(11): 10049–10057. 27934074 10.1021/acsnano.6b04695

[92] Zou Y , Wang Y , Xu S , Liu Y , Yin J , Lovejoy DB , Zheng M , Liang XJ , Park JB , Efremov YM , et al. Brain co-delivery of temozolomide and cisplatin for combinatorial glioblastoma chemotherapy. Adv Mater. 2022;34(33):2203958. 10.1002/adma.202203958 35738390

[93] Zhang H , Guan S , Wei T , Wang T , Zhang J , You Y , Wang Z , Dai Z . Homotypic membrane-enhanced blood-brain barrier crossing and glioblastoma targeting for precise surgical resection and photothermal therapy. J Am Chem Soc. 2023;145(10):5930–5940. 36867864 10.1021/jacs.2c13701

[94] Han D , Wang F , Qiao Z , Wang B , Zhang Y , Jiang Q , Liu M , Zhuang Y , An Q , Bai Y , et al. Neutrophil membrane-camouflaged nanoparticles alleviate inflammation and promote angiogenesis in ischemic myocardial injury. Bioact Mater. 2023;23:369–382. 36474655 10.1016/j.bioactmat.2022.11.016 PMC9706603

[95] Bi Y , Qian P , Su Z , Dai W , Xu F , Luo C . Construction of biomimetic camouflaged neutrophil membrane nanoparticles for precise delivery and augmented glioma cancer treatment. Process Biochem. 2024;145:229–242.

[96] Broekman ML , Maas SL , Abels ER , Mempel TR , Krichevsky AM , Breakefield XO . Multidimensional communication in the microenvirons of glioblastoma. Nat Rev Neurol. 2018;14(8):482–495. 29985475 10.1038/s41582-018-0025-8 PMC6425928

[97] Yin Y , Tang W , Ma X , Tang L , Zhang Y , Yang M , Hu F , Li G , Wang Y . Biomimetic neutrophil and macrophage dual membrane-coated nanoplatform with orchestrated tumor-microenvironment responsive capability promotes therapeutic efficacy against glioma. Chem Eng J. 2022;433:Article 133848.

[98] Dobbie MS , Hurst RD , Klein NJ , Surtees RA . Upregulation of intercellular adhesion molecule-1 expression on human endothelial cells by tumour necrosis factor-α in an in vitro model of the blood-brain barrier. Brain Res. 1999;830(2): 330–336. 10366690 10.1016/s0006-8993(99)01436-5

[99] Cheng VW , de Pennington N , Zakaria R , Larkin JR , Serres S , Sarkar M , Kirkman MA , Bristow C , Croal P , Plaha P , et al. VCAM-1-targeted MRI improves detection of the tumor-brain interface. Clin Cancer Res. 2022;28(11):2385–2396. 35312755 10.1158/1078-0432.CCR-21-4011 PMC9662863

[100] Mäenpää A , Kovanen PE , Anders P , Juha J , Tuomo T . Lymphocyte adhesion molecule ligands and extracellular matrix proteins in gliomas and normal brain: Expression of VCAM-1 in gliomas. Acta Neuropathol. 1997;94(3):216–225. 9292690 10.1007/s004010050696

[101] Lai J , Deng G , Sun Z , Peng X , Li J , Gong P , Zhang P , Cai L . Scaffolds biomimicking macrophages for a glioblastoma NIR-Ib imaging guided photothermal therapeutic strategy by crossing blood-brain barrier. Biomaterials. 2019;211:48–56. 31085358 10.1016/j.biomaterials.2019.04.026

[102] Xiao T , He M , Xu F , Fan Y , Jia B , Shen M , Wang H , Shi X . Macrophage membrane-camouflaged responsive polymer nanogels enable magnetic resonance imaging-guided chemotherapy/chemodynamic therapy of orthotopic glioma. ACS Nano. 2021;15(12):20377–20390. 34860014 10.1021/acsnano.1c08689

[103] Ma X , Kuang L , Yin Y , Tang L , Zhang Y , Fan Q , Wang B , Dong Z , Wang W , Yin T , et al. Tumor-antigen activated dendritic cell membrane-coated biomimetic nanoparticles with orchestrating immune responses promote therapeutic efficacy against glioma. ACS Nano. 2023;17(3):2341–2355. 36688797 10.1021/acsnano.2c09033

[104] Deng G , Peng X , Sun Z , Zheng W , Yu J , Du L , Chen H , Gong P , Zhang P , Cai L . Natural-killer-cell-inspired nanorobots with aggregation-induced emission characteristics for near-infrared-II fluorescence-guided glioma theranostics. ACS Nano. 2020;14(9):11452–11462. 32820907 10.1021/acsnano.0c03824

[105] Li Q , Shen J , Wu L , Lei S , Yang Y , Xu W , Hao K , Zhang Y , Kong F , Yang W , et al. Functional targeted therapy for glioma based on platelet membrane-coated nanogels. Cancer Nanotechnol. 2023;14(1):12.

[106] Li X , Qu B , Jin X , Hai L , Wu Y . Design, synthesis and biological evaluation for docetaxel-loaded brain targeting liposome with “lock-in” function. J Drug Target. 2014;22(3):251–261. 24313929 10.3109/1061186X.2013.865032

[107] Qu B , Li X , Guan M , Li X , Hai L , Wu Y . Design, synthesis and biological evaluation of multivalent glucosides with high affinity as ligands for brain targeting liposomes. Eur J Med Chem. 2014;72:110–118. 24361523 10.1016/j.ejmech.2013.10.007

[108] Peng Y , Zhao Y , Chen Y , Yang Z , Zhang L , Xiao W , Yang J , Guo L , Wu Y . Dual-targeting for brain-specific liposomes drug delivery system: Synthesis and preliminary evaluation. Bioorg Med Chem. 2018;26(16):4677–4686. 30098913 10.1016/j.bmc.2018.08.006

[109] Liu Q , Zhou L , Lu R , Yang C , Wang S , Hai L , Wu Y . Biotin and glucose co-modified multi-targeting liposomes for efficient delivery of chemotherapeutics for the treatment of glioma. Bioorg Med Chem. 2021;29:Article 115852. 33189509 10.1016/j.bmc.2020.115852

[110] Uchida Y , Ito K , Ohtsuki S , Kubo Y , Suzuki T , Terasaki T . Major involvement of Na+-dependent multivitamin transporter(SLC5A6/SMVT) in uptake of biotin and pantothenic acid by human brain capillary endothelial cells. J Neurochem. 2015;134(1):97–112. 25809983 10.1111/jnc.13092

[111] Veszelka S , Meszaros M , Kiss L , Kota Z , Pali T , Hoyk Z , Bozso Z , Fulop L , Toth A , Rakhely G , et al. Biotin and glutathione targeting of solid nanoparticles to cross human brain endothelial cells. Curr Pharm Des. 2017;23(28): 4198–4205. 28748755 10.2174/1381612823666170727144450

[112] Zhao P , Tian Y , Lu Y , Zhang J , Tao A , Xiang G , Liu Y . Biomimetic calcium carbonate nanoparticles delivered IL-12 mRNA for targeted glioblastoma sono-immunotherapy by ultrasound-induced necroptosis. J Nanobiotechnol. 2022;20(1):525. 10.1186/s12951-022-01731-z PMC9741778 36496387

[113] Miura Y , Takenaka T , Toh K , Wu S , Nishihara H , Kano MR , Ino Y , Nomoto T , Matsumoto Y , Koyama H , et al. Cyclic RGD-linked polymeric micelles for targeted delivery of platinum anticancer drugs to glioblastoma through the blood-brain tumor barrier. ACS Nano. 2013;7(10):8583–8592. 24028526 10.1021/nn402662d

[114] Zhang W , Chen X , Ding D , Zhang G , Zhu Z , Yang X , Li M , Liang L , Shi X , Wang T , et al. Real-time in vivo imaging reveals specific nanoparticle target binding in a syngeneic glioma mouse model. Nanoscale. 2022;14(15):5678–5688. 35195122 10.1039/d1nr07591h

[115] Mo J , Chen X , Li M , Liu W , Zhao W , Lim LY , Tilley RD , Gooding JJ , Li Q . Upconversion nanoparticle-based cell membrane-coated cRGD peptide bioorthogonally labeled nanoplatform for glioblastoma treatment. ACS Appl Mater Interfaces. 2022;14(44):49454–49470. 10.1021/acsami.2c11284 36300690

[116] Yang T , Zhang N , Liu Y , Yang R , Wei Z , Liu F , Song D , Wang L , Wei J , Li Y , et al. Nanoplatelets modified with RVG for targeted delivery of miR-375 and temozolomide to enhance gliomas therapy. J Nanobiotechnol. 2024;22(1):623. 10.1186/s12951-024-02895-6 PMC11476726 39402578

[117] Ma J , Dai L , Yu J , Cao H , Bao Y , Hu J , Zhou L , Yang J , Sofia A , Chen H , et al. Tumor microenvironment targeting system for glioma treatment via fusion cell membrane coating nanotechnology. Biomaterials. 2023;295:Article 122026. 36731366 10.1016/j.biomaterials.2023.122026

[118] Banks WA , Tschöp M , Robinson SM , Heiman ML . Extent and direction of ghrelin transport across the blood-brain barrier is determined by its unique primary structure. J Pharmacol Exp Ther. 2002;302(2):822–827. 12130749 10.1124/jpet.102.034827

[119] Chen Y-C , Chiang C-F , Chen L-F , Liao S-C , Hsieh W-Y , Lin W-L . Polymersomes conjugated with des-octanoyl ghrelin for the delivery of therapeutic and imaging agents into brain tissues. Biomaterials. 2014;35(6):2051–2065. 24315575 10.1016/j.biomaterials.2013.11.051

[120] Chen Y-C , Chiang C-F , Chen L-F , Liang P-C , Hsieh W-Y , Lin W-L . Polymersomes conjugated with des-octanoyl ghrelin and folate as a BBB-penetrating cancer cell-targeting delivery system. Biomaterials. 2014;35(13):4066–4081. 24513319 10.1016/j.biomaterials.2014.01.042

[121] Niu J , Wang A , Ke Z , Zheng Z . Glucose transporter and folic acid receptor-mediated Pluronic P105 polymeric micelles loaded with doxorubicin for brain tumor treating. J Drug Target. 2014;22(8):712–723. 24806516 10.3109/1061186X.2014.913052

[122] Gao J-Q , Lv Q , Li L-M , Tang X-J , Li F-Z , Hu Y-L , Han M . Glioma targeting and blood-brain barrier penetration by dual-targeting doxorubincin liposomes. Biomaterials. 2013;34(22):5628–5639. 23628475 10.1016/j.biomaterials.2013.03.097

[123] Onoe S . Development of molecular probes for spatio-temporal analysis of in vivo tumor with photoacoustic imaging. Yakugaku zasshi. 2016;136(3):491–498. 26935092 10.1248/yakushi.15-00249

[124] Sarkaria JN , Hu LS , Parney IF , Pafundi DH , Brinkmann DH , Laack NN , Giannini C , Burns TC , Kizilbash SH , Laramy JK , et al. Is the blood-brain barrier really disrupted in all glioblastomas? A critical assessment of existing clinical data. Neuro Oncol. 2018;20(2):184–191. 29016900 10.1093/neuonc/nox175 PMC5777482

[125] Chen C , Duan Z , Yuan Y , Li R , Pang L , Liang J , Xu X , Wang J . Peptide-22 and cyclic RGD functionalized liposomes for glioma targeting drug delivery overcoming BBB and BBTB. ACS Appl Mater Interfaces. 2017;9(7):5864–5873. 28128553 10.1021/acsami.6b15831

[126] Guan J , Jiang Z , Wang M , Liu Y , Liu J , Yang Y , Ding T , Lu W , Gao C , Qian J , et al. Short peptide-mediated brain-targeted drug delivery with enhanced immunocompatibility. Mol Pharm. 2019;16(2):907–913. 30666875 10.1021/acs.molpharmaceut.8b01216

[127] Farshbaf M , Khosroushahi AY , Mojarad-Jabali S , Zarebkohan A , Valizadeh H , Walker PR . Cell surface GRP78: An emerging imaging marker and therapeutic target for cancer. J Control Release. 2020;328:932–941. 33129921 10.1016/j.jconrel.2020.10.055

[128] Kang BR , Yang S-H , Chung B-R , Kim W , Kim Y . Cell surface GRP78 as a biomarker and target for suppressing glioma cells. Sci Rep. 2016;6(1):34922. 27713511 10.1038/srep34922 PMC5054676

[129] Chen M , Cui Y , Hao W , Fan Y , Zhang J , Liu Q , Jiang M , Yang Y , Wang Y , Gao C . Ligand-modified homologous targeted cancer cell membrane biomimetic nanostructured lipid carriers for glioma therapy. Drug Deliv. 2021;28(1):2241–2255. 34668811 10.1080/10717544.2021.1992038 PMC8530486

[130] Fan Y , Cui Y , Hao W , Chen M , Liu Q , Wang Y , Yang M , Li Z , Gong W , Song S , et al. Carrier-free highly drug-loaded biomimetic nanosuspensions encapsulated by cancer cell membrane based on homology and active targeting for the treatment of glioma. Bioact Mater. 2021;6(12):4402–4414. 33997516 10.1016/j.bioactmat.2021.04.027 PMC8111096

[131] Ran D , Mao J , Zhan C , Xie C , Ruan H , Ying M , Zhou J , Lu W-L , Lu W . D-Retroenantiomer of quorum-sensing peptide-modified polymeric micelles for brain tumor-targeted drug delivery. ACS Appl Mater Interfaces. 2017;9(31):25672–25682. 28548480 10.1021/acsami.7b03518

[132] Hao W , Cui Y , Fan Y , Chen M , Yang G , Wang Y , Yang M , Li Z , Gong W , Yang Y , et al. Hybrid membrane-coated nanosuspensions for multi-modal anti-glioma therapy via drug and antigen delivery. J Nanobiotechnololgy. 2021;19:378. 10.1186/s12951-021-01110-0 PMC8606100 34801032

[133] Xie Y , He L , Lugano R , Zhang Y , Cao H , He Q , Chao M , Liu B , Cao Q , Wang J , et al. Key molecular alterations in endothelial cells in human glioblastoma uncovered through single-cell RNA sequencing. JCI Insight. 2021;6(15):e150861. 34228647 10.1172/jci.insight.150861 PMC8410070

[134] Zhang P , Chen D , Li L , Sun K . Charge reversal nano-systems for tumor therapy. J Nanobiotechnololgy. 2022;20(1):31. 10.1186/s12951-021-01221-8 PMC8751315 35012546

[135] Testa E , Palazzo C , Mastrantonio R , Viscomi MT . Dynamic interactions between tumor cells and brain microvascular endothelial cells in glioblastoma. Cancers. 2022;14(13):3128. 35804908 10.3390/cancers14133128 PMC9265028

[136] Chang Y , Cai X , Syahirah R , Yao Y , Xu Y , Jin G , Bhute VJ , Torregrosa-Allen S , Elzey BD , Won Y-Y , et al. CAR-neutrophil mediated delivery of tumor-microenvironment responsive nanodrugs for glioblastoma chemo-immunotherapy. Nat Commun. 2023;14(1):2266. 37080958 10.1038/s41467-023-37872-4 PMC10119091

[137] Ananda S , Nowak AK , Cher L , Dowling A , Brown C , Simes J , Rosenthal MA , Cooperative Trials Group for Neuro-Oncology(COGNO) . Phase 2 trial of temozolomide and pegylated liposomal doxorubicin in the treatment of patients with glioblastoma multiforme following concurrent radiotherapy and chemotherapy. J Clin Neurosci. 2011;18(11):1444–1448. 21813279 10.1016/j.jocn.2011.02.026

[138] Birngruber T , Raml R , Gladdines W , Gatschelhofer C , Gander E , Ghosh A , Kroath T , Gaillard PJ , Pieber TR , Sinner F . Enhanced doxorubicin delivery to the brain administered through glutathione PEGylated liposomal doxorubicin(2B3-101) as compared with generic Caelyx®/Doxil®—A cerebral open flow microperfusion pilot study. J Pharm Sci. 2014;103(7):1945–1948. 24801480 10.1002/jps.23994

[139] Maussang D , Rip J , van Kregten J , van den Heuvel A , van der Pol S , van der Boom B , Reijerkerk A , Chen L , de Boer M , Gaillard P , et al. Glutathione conjugation dose-dependently increases brain-specific liposomal drug delivery in vitro and in vivo. Drug Discov Today Technol. 2016;20:59–69. 27986226 10.1016/j.ddtec.2016.09.003

[140] Pan Y , Xu C , Deng H , You Q , Zhao C , Li Y , Gao Q , Akakuru OU , Li J , Zhang J , et al. Localized NIR-II laser mediated chemodynamic therapy of glioblastoma. Nano Today. 2022;43:Article 101435.

[141] Fan Y , Hao W , Cui Y , Chen M , Chu X , Yang Y , Wang Y , Gao C . Cancer cell membrane-coated nanosuspensions for enhanced chemotherapeutic treatment of glioma. Molecules. 2021;26(16):5103. 34443689 10.3390/molecules26165103 PMC8400986

[142] Ruan W , Jiao M , Xu S , Ismail M , Xie X , An Y , Guo H , Qian R , Shi B , Zheng M . Brain-targeted CRISPR/Cas9 nanomedicine for effective glioblastoma therapy. J Control Release. 2022;351:739–751. 36174804 10.1016/j.jconrel.2022.09.046

[143] Li B , Chen X , Qiu W , Zhao R , Duan J , Zhang S , Pan Z , Zhao S , Guo Q , Qi Y , et al. Synchronous disintegration of ferroptosis defense axis via engineered exosome-conjugated magnetic nanoparticles for glioblastoma therapy. Adv Sci. 2022;9(17):Article e2105451. 10.1002/advs.202105451 PMC9189685 35508804

[144] Li C , Guan N , Liu F . T7 peptide-decorated exosome-based nanocarrier system for delivery of Galectin-9 siRNA to stimulate macrophage repolarization in glioblastoma. J Neurooncol. 2023;162(1):93–108. 36854924 10.1007/s11060-023-04257-y

[145] Mojarad-Jabali S , Farshbaf M , Hemmati S , Sarfraz M , Motasadizadeh H , Mojarrad JS , Atyabi F , Zakeri-Milani P , Valizadeh H . Comparison of three synthetic transferrin mimetic small peptides to promote the blood-brain barrier penetration of vincristine liposomes for improved glioma targeted therapy. Int J Pharm. 2022;613:Article 121395. 34933080 10.1016/j.ijpharm.2021.121395

[146] Kang T , Jiang M , Jiang D , Feng X , Yao J , Song Q , Chen H , Gao X , Chen J . Enhancing glioblastoma-specific penetration by functionalization of nanoparticles with an iron-mimic peptide targeting transferrin/transferrin receptor complex. Mol Pharm. 2015;12(8):2947–2961. 26149889 10.1021/acs.molpharmaceut.5b00222

[147] Kim S-S , Rait A , Kim E , Pirollo KF , Nishida M , Farkas N , Dagata JA , Chang EH . A nanoparticle carrying the p53 gene targets tumors including cancer stem cells, sensitizes glioblastoma to chemotherapy and improves survival. ACS Nano. 2014;8(6):5494–5514. 24811110 10.1021/nn5014484 PMC4076028

[148] Kang S , Duan W , Zhang S , Chen D , Feng J , Qi N . Muscone/RI7217 co-modified upward messenger DTX liposomes enhanced permeability of blood-brain barrier and targeting glioma. Theranostics. 2020;10(10):4308–4322. 32292496 10.7150/thno.41322 PMC7150489

[149] Su Z , Xing L , Chen Y , Xu Y , Yang F , Zhang C , Ping Q , Xiao Y . Lactoferrin-modified poly(ethylene glycol)-grafted BSA nanoparticles as a dual-targeting carrier for treating brain gliomas. Mol Pharm. 2014;11(6):1823–1834. 24779677 10.1021/mp500238m

[150] Song M-M , Xu H-L , Liang J-X , Xiang H-H , Liu R , Shen Y-X . Lactoferrin modified graphene oxide iron oxide nanocomposite for glioma-targeted drug delivery. Mater Sci Eng C Mater Biol Appl. 2017;77:904–911. 28532109 10.1016/j.msec.2017.03.309

[151] Li Y , Pan Y , Wang Y , Jiang Z , Akakuru OU , Li M , Zhang X , Yuan B , Xing J , Luo L , et al. A D-peptide ligand of neuropeptide Y receptor Y1 serves as nanocarrier traversing of the blood brain barrier and targets glioma. Nano Today. 2022;44:Article 101465.

[152] Elechalawar CK , Bhattacharya D , Ahmed MT , Gora H , Sridharan K , Chaturbedy P , Sinha SH , Jaggarapu MMCS , Narayan KP , Chakravarty S , et al. Dual targeting of folate receptor-expressing glioma tumor-associated macrophages and epithelial cells in the brain using a carbon nanosphere-cationic folate nanoconjugate. Nanoscale Adv. 2019;1(9):3555–3567. 36133563 10.1039/c9na00056a PMC9417975

[153] Sun R , Liu M , Xu Z , Song B , He Y , Wang H . Silicon-based nanoprobes cross the blood-brain barrier for photothermal therapy of glioblastoma. Nano Res. 2022;15(8):7392–7401.

[154] Suzuki K , Miura Y , Mochida Y , Miyazaki T , Toh K , Anraku Y , Melo V , Liu X , Ishii T , Nagano O , et al. Glucose transporter 1-mediated vascular translocation of nanomedicines enhances accumulation and efficacy in solid tumors. J Control Release. 2019;301: 28–41. 30844476 10.1016/j.jconrel.2019.02.021

[155] Bhunia S , Vangala V , Bhattacharya D , Ravuri HG , Kuncha M , Chakravarty S , Sistla R , Chaudhuri A . Large amino acid transporter 1 selective liposomes of l-DOPA functionalized amphiphile for combating glioblastoma. Mol Pharm. 2017;14(11):3834–3847. 28958145 10.1021/acs.molpharmaceut.7b00569

