# Supplementary material for: Combined Strategies for Nanodrugs Noninvasively Overcoming the Blood–Brain Barrier and Actively Targeting Glioma Lesions
Source: Biomater Res. 2025 Feb 5;29:0133. doi: 10.34133/bmr.0133 (PMC11794768; doi:10.34133/bmr.0133)
Supplement: Supplementary 1 — Figs. S1 to S5 Tables S1 and S2 References [140,141] [file bmr.0133.f1.docx]

***<Supplemental Materials>***

Combined Strategies for Nanodrugs Non-invasively Overcoming BBB and Actively Targeting Glioma Lesions

Yuanyuan Liu^1^, Haigang Wu^2^*, Gaofeng Liang^1^*

1. College of Basic Medicine and Forensic Medicine, Henan University of Science and Technology, Luoyang, Henan Province, 471000, China
2. Henan Key Laboratory of Brain Targeted Bio-nanomedicine, School of Life Sciences & School of Pharmacy, Henan University, Kaifeng, Henan Province, 475004, China

*Corresponding authors.

*E-mail* *addresses*: whg_2018@henu.edu.cn (H.W.), lgfeng990448@haust.edu.cn (G.L.)


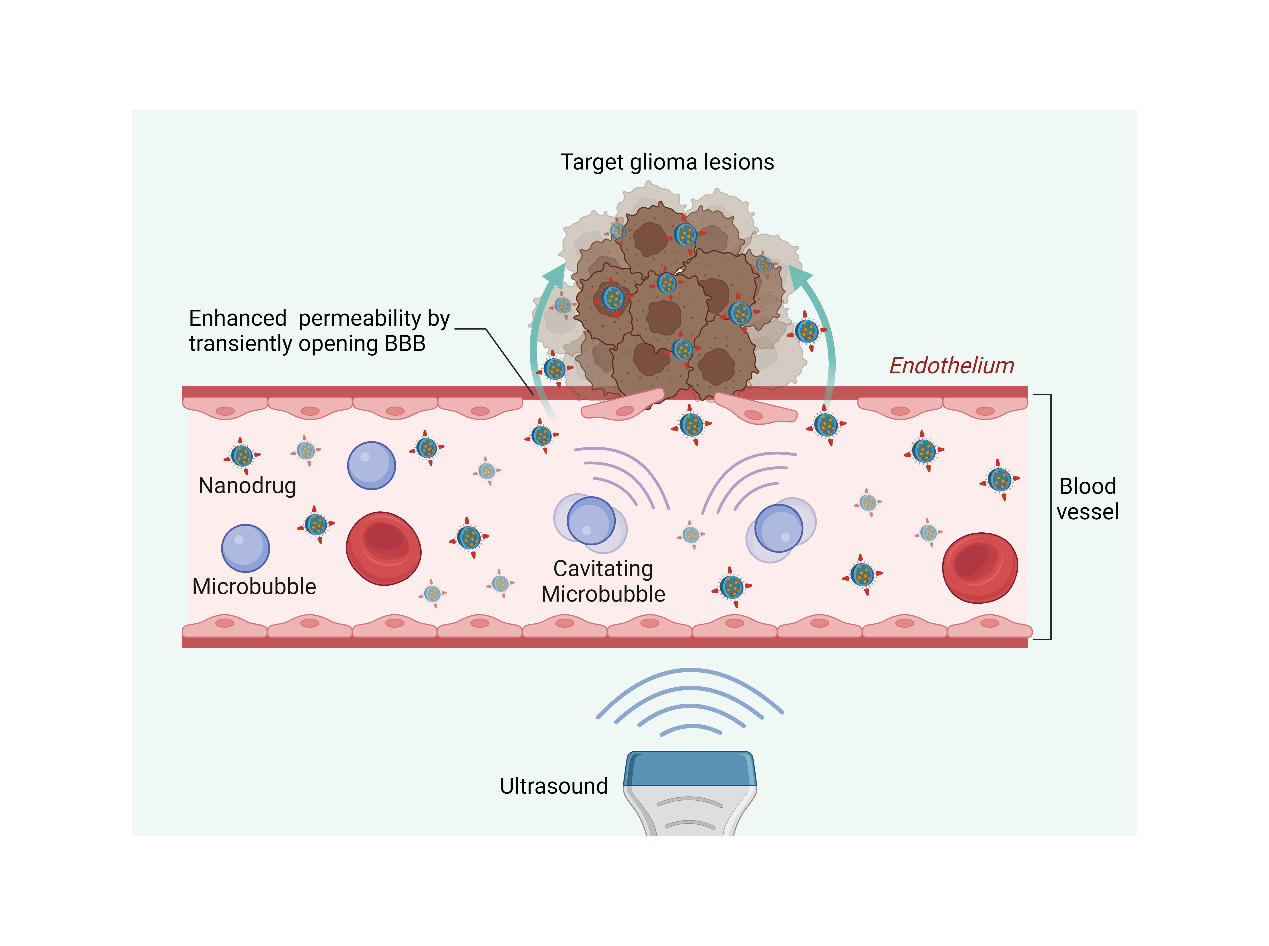


**Fig. S1.** The scheme illustrating the BBB opening under the function of focused ultrasound and microbubbles. The microbubbles cavitate under ultrasound irradiation, resulting in BBB structures of blood vessels being stretched and expanded, and hence transiently opening of the BBB. Glioma-targeting decorated nanodrugs can passively pass the BBB and actively accumulate in glioma lesions. The schematic diagram is adapted based on the BioRender template “Lipid-Based Microbubbles (MBs) as Ultrasound-Based Drug Delivery System”. (Biorender)

**Fig. S2.** Tumor treated with Lipo-Dox (A) and AP-1 Lipo-Dox (B), and the decoration of AP-1 helps Lipo-Dox actively target to tumor site. (C) Pulsed HIFU with microbubbles significantly enhances the delivery of AP-1 Lipo-Dox to the tumor site. (D) Analysis of tumor size receiving the different treatments. (E) Kaplan-Meier survival curves of mice with the different treatments. Reproduced from [32] with permission from the Elsevier, Copyright 2012.

**Fig. S3.** (A) Construction of MPM@P NGs. (B) The mechanism of biomimetic MPM@P NGs crosses the BBB and penetrates brain parenchyma for MR imaging-guided combination therapy. (C) Schematic diagram of the in vitro BBB model. (D) Percentage penetration of Mn/Pt in the inferior vena cava. Representative MR images (E) of glioma-bearing mice receiving different treatments and the quantitative relative tumor volume (F) and body weight (G). Reproduced from [112] with permission from the American Chemical Society, Copyright 2021.

**Fig. S4.** (A) The process of des-octanoyl ghrelin and folate co-decorated and Dox-loaded polymersome (GFP-D) crossing the BBB and targeting glioma cells. (B) Fluorescence imaging of C6 glioma cells growing in the underlying BBB model after incubation with different materials. (C) Survival curves of the glioma-bearing mice under different treatments. Reproduced from [139] with permission from the Elsevier, Copyright 2014.


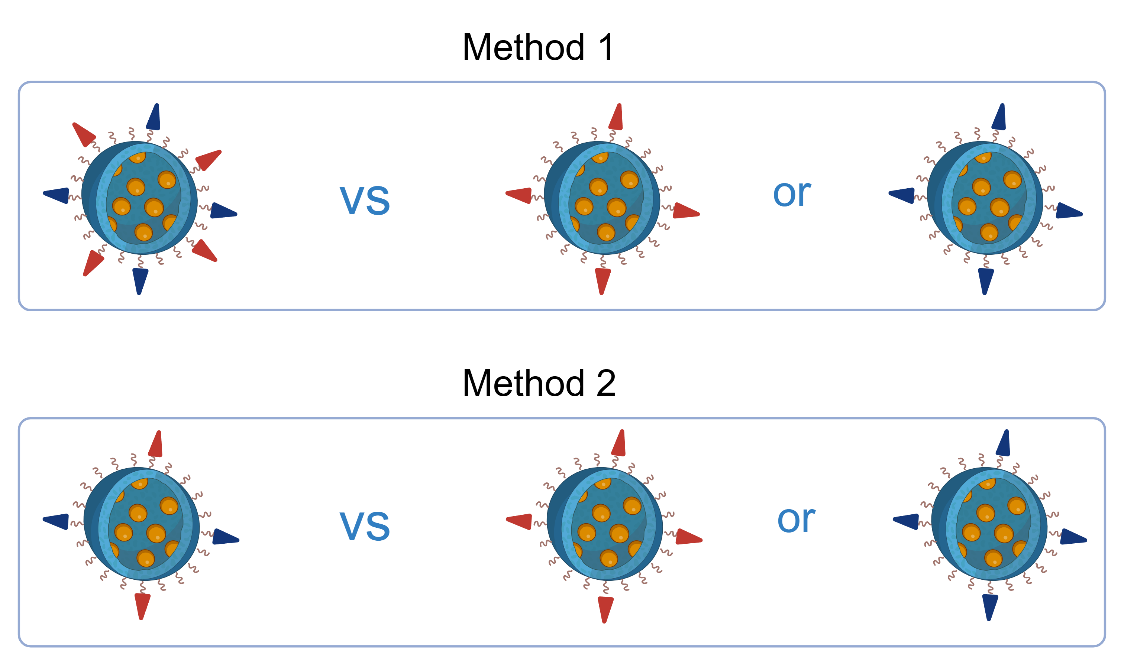


**Fig. S5.** Scheme for two comparison methods between dual-targeted and single-targeted modified nanomedicines. (Biorender)

**Table S1**. The clinical strategies for treating gliomas with different WHO grades (https://www.nccn.org/).

| Grade | Primary treatment | Adjuvant treatment |
| --- | --- | --- |
| Low-grade (I/ II) | Surgery | Low risk: clinical trial or kept under observation |
|  |  | High risk: radiation, chemotherapy (PCV or temozolomide) |
| High-grade (III/ IV) | Surgery | The clinical trial is first recommended. If it isn’t possible or available, radiation, chemotherapy (PCV or temozolomide) or other therapies like targeted therapy will be administrated. |

**Table S2**. The utilization of nanodrugs featuring dual-targeting functionality mediated by type cell membrane camouflage holds promising applications in the treatment of glioma.

| **Type of cell** | **CM fragment sources** | **Nanocarrier** | **Used drug**  **“Therapeutic strategy”** | **Cell model** | **Ref.** |
| --- | --- | --- | --- | --- | --- |
| Glioma cell membrane  (GCM) | C6 or U87MG | Vitamin E succinate-grafted ε-polylysine polymer | TRAIL plasmid  “TT” | C6 or U87MG | [107] |
|  | U251 R or U87MG | acetalated dextran (Ac-DEX) | TMZ and CDDP  “CT” | U251 R or U87MG | [108] |
|  | U87MG | gold nanorods (AuNRs) | AuNRs  “Raman guided resection/PTT” | U87MG | [109] |
|  | U87MG | CuFeSe2 | lactate oxidase  “chemodynamic therapy (CDT)” | U87MG | [110] |
|  | C6 | nanosuspension | 10-hydroxycamptothecin  “CT” | C6 | [111] |
| Immune cell membrane  (ICM) | Macrophage | poly(N-vinylcaprolactam) | manganese dioxide (MnO_2_) + cisplatin  “Imaging + CT + CDT” | C6 | [112] |
|  |  | DSPE-PEG | IR-792  “Imaging + PTT” | U87L | [113] |
|  | Tumor-antigen activated dendritic cell | PLGA | RAPA + active immune cells  “CT + IT” | C6 | [114] |
|  | Natural kill cell | liposome | AIE-active polymeric endoskeleton (PBPTV)  “Imaging + PTT” | U87MG | [115] |
| Platelet membrane (PM) | Platelet | formylphenylboronic acid/ethylene imine polymer (nanogel) | Doxorubicin  “CT” | C6 | [116] |
